# Allele-Selective Transcriptome Recruitment to Polysomes Primed for Translation: Protein-Coding and Noncoding RNAs, and RNA Isoforms

**DOI:** 10.1371/journal.pone.0136798

**Published:** 2015-09-02

**Authors:** Roshan Mascarenhas, Maciej Pietrzak, Ryan M. Smith, Amy Webb, Danxin Wang, Audrey C. Papp, Julia K. Pinsonneault, Michal Seweryn, Grzegorz Rempala, Wolfgang Sadee

**Affiliations:** 1 Center for Pharmacogenomics, College of Medicine, The Ohio State University Wexner Medical Center, Columbus, Ohio, United States of America; 2 Division of Biostatistics, College of Public Health, The Ohio State University, Columbus, Ohio, United States of America; 3 Department of Biomedical Informatics, College of Medicine, The Ohio State University Wexner Medical Center, Columbus, Ohio, United States of America; 4 Department of Mathematics and Computer Science, University of Lodz, Lodz, Poland; 5 Mathematical Biosciences Institute, The Ohio State University, Columbus, Ohio, United States of America; 6 Department of Medical Genetics, College of Medicine, The Ohio State University Wexner Medical Center, Columbus, Ohio, United States of America; The John Curtin School of Medical Research, AUSTRALIA

## Abstract

mRNA translation into proteins is highly regulated, but the role of mRNA isoforms, noncoding RNAs (ncRNAs), and genetic variants remains poorly understood. mRNA levels on polysomes have been shown to correlate well with expressed protein levels, pointing to polysomal loading as a critical factor. To study regulation and genetic factors of protein translation we measured levels and allelic ratios of mRNAs and ncRNAs (including microRNAs) in lymphoblast cell lines (LCL) and in polysomal fractions. We first used targeted assays to measure polysomal loading of mRNA alleles, confirming reported genetic effects on translation of *OPRM1* and *NAT1*, and detecting no effect of rs1045642 (*3435C>T*) in *ABCB1* (MDR1) on polysomal loading while supporting previous results showing increased mRNA turnover of the *3435T* allele. Use of high-throughput sequencing of complete transcript profiles (RNA-Seq) in three LCLs revealed significant differences in polysomal loading of individual RNA classes and isoforms. Correlated polysomal distribution between protein-coding and non-coding RNAs suggests interactions between them. Allele-selective polysome recruitment revealed strong genetic influence for multiple RNAs, attributable either to differential expression of RNA isoforms or to differential loading onto polysomes, the latter defining a direct genetic effect on translation. Genes identified by different allelic RNA ratios between cytosol and polysomes were enriched with published expression quantitative trait loci (eQTLs) affecting RNA functions, and associations with clinical phenotypes. Polysomal RNA-Seq combined with allelic ratio analysis provides a powerful approach to study polysomal RNA recruitment and regulatory variants affecting protein translation.

## Introduction

**Fig 1:**
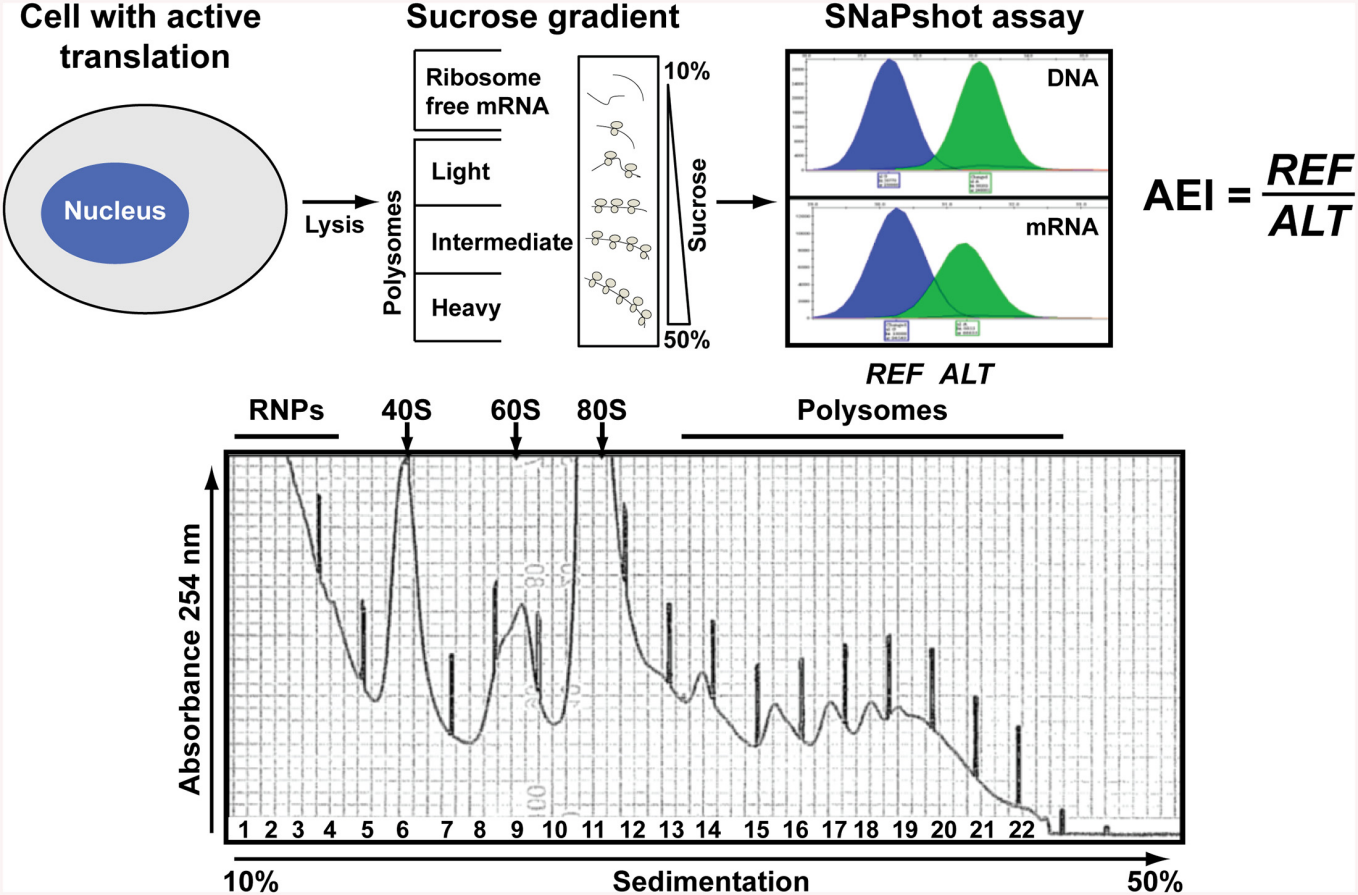
Measuring allelic mRNA occupancy on polysomes. The flowchart depicts the strategy used to fractionate cellular components for RNA analysis and to determine Allelic Expression Imbalance (AEI), defined as a ratio of reference allele (REF) to alternative allele (ALT). Chromatographic peaks obtained from SNaPshot [25] represent relative allelic abundance in gDNA and mRNA (as cDNA). Significant deviation from unity (after normalization to allelic ratios of gDNA or plasmid DNA) indicates unequal polysomal occupancy of allelic mRNA. RNPs represent cytosolic mRNA either polysome-free or bound to ribonucleotide particles.

Extensive studies have revealed molecular features of mRNAs enabling polysomal loading and translation [1, 2]. Protein expression was proposed to be regulated with near equal magnitude at the level of transcription and translation [3]. Protein levels can increase up to 20-fold without corresponding alterations in mRNA abundance, and mRNA levels can increase up to 30-fold without reflection on protein levels [4–6]. Correlations between cellular mRNA and protein levels can be drastically improved by measuring mRNAs bound to polysomes, a series of ribosomes held together with a strand of messenger RNA undergoing active translation (Fig 1) [6, 7]. Differential recruitment of mRNAs and isoforms to polysomes contribute a critical layer of regulation involving sequence specific regulatory events [8, 9]. Moreover, cell stimulation with EGF has revealed strong uncoupling of the transcriptome and the translatome, through rapid recruitment of mRNAs onto polysomes rather than new transcription [8, 9]. These studies, using sequencing (RNA-Seq) and hybridization arrays, respectively, have employed poly-*dT* for cDNA synthesis, thereby lacking ability to detect RNAs without poly-*A* tails and small RNAs.

Whereas loading of protein-coding mRNAs onto translating polysomes has been studied extensively, less is known about other classes of RNAs, including antisense and pseudogene RNAs, long (intergenic) noncoding RNAs (l(i)ncRNAs), and short RNAs such as microRNAs [10]. Therefore, one of the goals of this study was to perform an initial survey of all RNA classes associated with polysomes. To detect all RNAs and their isoforms, we have performed RNA-Seq with random hexamer-priming and separate analyses of short RNAs, encompassing the entire transcriptome, using a procedure that depletes ribosomal RNA.

A second focus of this study was to develop an approach for detecting genetic variants altering polysomal RNA loading. The human genome contains numerous variants affecting transcription [11] and RNA processing events [12, 13]. Since mRNA and protein levels are often poorly correlated [14–17], mRNA expression quantitative trait loci (eQTLs) sometimes fail to reflect corresponding changes in protein expression [18, 19] and only partially overlap with protein eQTLs [20]. Comprehensive studies of genetic factors acting specifically on polysomal loading and translation are sparse. Targeted molecular studies examining the effects of genetic variants on translation typically involve reporter gene assays; however, results obtained in cell culture system, may not reflect native tissue conditions [21]. A genome-wide mRNA analysis in cytosol and polysomes of lymphoblast cell lines (LCLs), with hybridization microarray methods combined with genome-wide single nucleotide polymorphism (SNP) assays, revealed numerous distinct eQTLs, suggesting pervasive genetic effects on translation [7]. At the post-transcriptional level, genetic factors could directly affect polysomal loading, or alter the formation of mRNA isoforms with differential access to polysomes. Both processes can be readily measured with mRNA microarray analysis.

A novel approach used here is to measure differential polysome loading of RNAs and their isoforms, coupled with allele-specific measurements, to assess the impact of genetic variation on translation. Differences in the allelic RNA ratios between cytosolic and polysomal extracts reveal the presence of regulatory factors that determine polysomal loading. The vast majority of genes present multiple RNA isoforms with different 3′ and 5′ UTRs and alternative splicing events, or undergoing RNA editing. To resolve these processes we employed full transcriptome sequencing (RNA-Seq) in LCLs to measure differential loading of all RNAs and their isoforms onto translating ribosomes. RNA-Seq further provides estimates of allelic ratios at heterozygous SNPs [22], revealing regulatory variants affecting transcription, RNA processing, and translation.

To test the utility of allelic RNA ratio analysis comparing total cellular to polysomal RNAs, we first employed a targeted gene approach investigating the effects on polysomal loading exerted by regulatory variants known to alter translation (*OPRM1* (*118 A>G*; rs1799971) and NAT1*10 (rs1057126)) [23, 24]. *ABCB1* (multidrug resistance 1 polypeptide, MDR1) was also included to test two proposed alternative mechanisms of post-transcriptional regulation. The synonymous SNP *3435C>T* (rs1045642) had been shown to alter mRNA stability [25], while another study suggested that usage of the rare codon introduced by *3435T* reduces translation [26], an event affecting polysome interactions, and thereby, could alter allelic ratios in the polysomes.

Here we report exploratory study analyzing the entire cellular transcriptome in comparison to the translatome (RNAs on polysomes). We also present a new approach to discover regulatory polymorphisms affecting translation, by measuring changes in allelic RNA ratios upon loading onto translating polysomes.

## Results

### Targeted measurements of polysomal allelic mRNA ratios in cell cultures

RNA fractions were recovered from total cytoplasmic lysate (cytosol) and from polysomal fractions (Fig 1), converted to cDNA, and analyzed by qRT-PCR. Spiking of the cytosolic and polysome fractions with luciferase mRNAs served as internal control. Target mRNAs were amplified with low cycle RT-PCR (non-saturating) from eluate fractions and the amplicons analyzed by gel electrophoresis, showing distributions of individual mRNAs across the gradient (S1A–S1C Fig). We then used qRT-PCR to determine the differences in cycle threshold (ΔCt) values relative to luciferase, yielding distinct profiles for *OPRM1*, *NAT1*, and *ABCB1* mRNAs across polysomal fractions (S1D–S1F Fig), consistent with previous findings [8, 9]. We then measured allelic mRNA ratios in cytosol and polysomal fraction to determine differential loading for each allele.

#### A. *OPRM1 118A>G* (rs1799971) affects both expression of cytoplasmic RNA alleles and polysome loading

**Fig 2:**
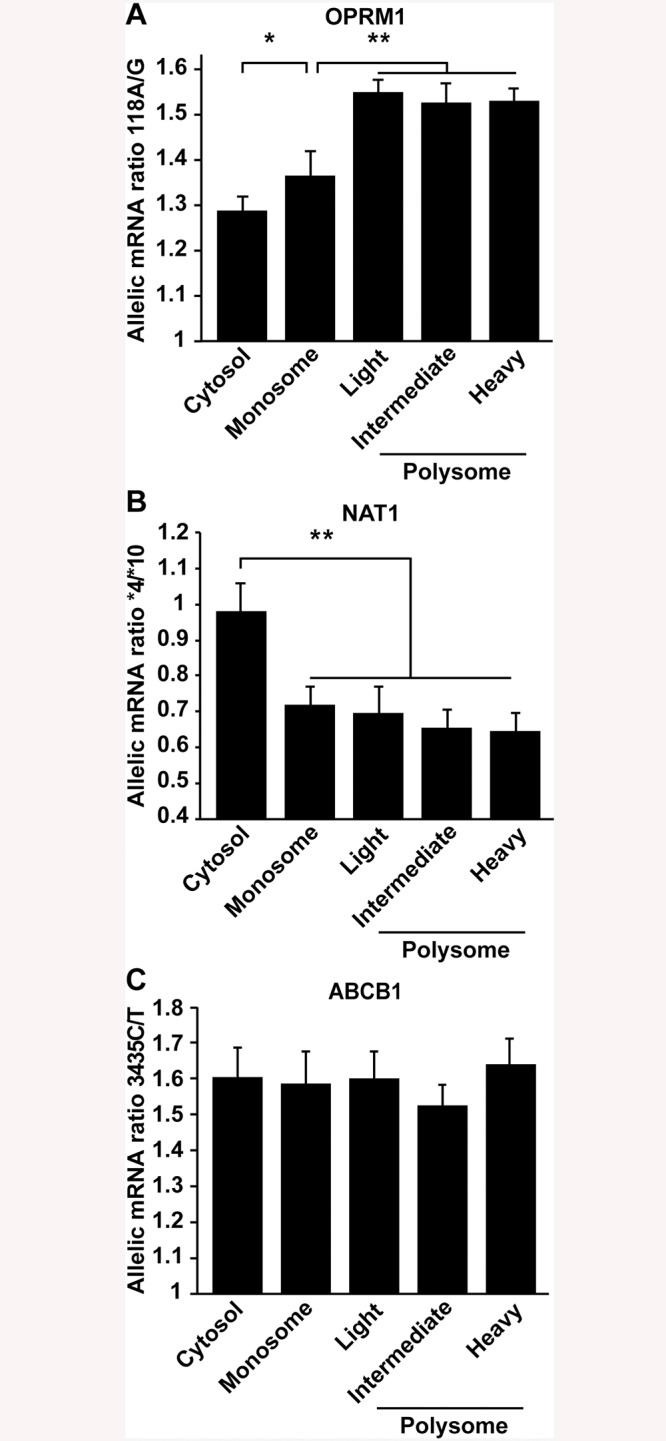
Allelic mRNA expression measurement in polysomes of transfected cell lines and cells with native expression of *NAT1* and *ABCB1*. **A.** Effect of *OPRM1 118A>G* polymorphism on cytoplasmic mRNA expression and polysome loading; an *A>G* ratio >1 indicates reduced levels of the minor *G* allele relative to the *A* allele. Equal amounts of *OPRM1* plasmids containing *118A* and *G* were co-transfected into CHO cells. The *118A/G* allelic ratios, measured with SNaPshot, represent the mean ± s.(n = 6), *p<0.049 for monosome *versus* cytoplasmic and **0.003 for monosome *versus* polysomes, two-tailed Student’s *t*-test. These profiles are representative of results from 3 independent cultures. **B-C.** AEI measurement in polysomes of lymphoblastoid cells with native expression of *NAT1* and *ABCB1*. LCL (1 x 10^7^ cells) heterozygous for *4/*10 were selected for polysome preparation. N-acetyltransferase 1 (NAT1) *10 allelic mRNA increased polysomal loading (lower *4/*10 ratios, *p*<0.01, corrected for gDNA ratios (B). Ribosomal occupancy of ABCB1 (MDR1) *3435C>T* allelic mRNA. *ABCB1 3435C>T* allelic mRNA ratios (C). Reduced *3435C/T* ratios demonstrated a significant reduction of the *3435T* allele in the cytoplasmic mRNA extract, with no significant difference in any gradient fraction. These profiles are representative of gradients done with extracts from 3 independent cultures (mean ± s.d., n = 6).

Equal amounts of plasmids carrying the entire *OPRM1* coding region with either the *118A* or the *118G* allele were transfected into CHO cells. As the *OPRM1 118G* allele had been shown to reduce both the overall *OPRM1* RNA expression and translational efficiency relative to the *118A* allele, measured by luciferase assays [23], we expected to observe increasingly greater allelic *A/G* ratios from cytoplasmic lysates to the polysomal fractions, reflecting preferential loading of *118A*. Accordingly, the normalized allelic mRNA ratios for *118A/G* was 1.3 in cytoplasmic lysate (n = 6, p<0.01), while allelic *A/G* ratios increased to 1.5–1.6-fold in polysomes, significantly greater than the cytosolic allelic mRNA ratio (n = 6, p<0.01) (Fig 2A), consistent with the proposed dual mechanism by which *118G* reduces OPRM1 protein expression [23].

#### B. N-acetyltransferase 1 NAT1*10 (rs1057126) increases polysomal mRNA loading

NAT1 is natively expressed in LCLs at levels sufficient for analysis. The NAT1*10 3′ UTR SNP had been shown to enhance translation in LCLs without affecting total cytoplasmic mRNA content [24], suggesting enhanced polysomal loading of the *10 allele *versus* the *4 wild-type allele. This hypothesis was tested in LCL sample GM07341, which is heterozygous for the NAT1*4/*10 alleles. Consistent with a lack of effect of NAT1*10 on transcription and mRNA processing, the allelic mRNA ratio NAT1*4/*10 in the cytoplasmic lysate did not differ from unity (0.98 ±0.08, p-value ≤ 0.05, n = 6) (Fig 2B). In contrast, monosomes and polysome fractions displayed allelic *4/*10 mRNA ratios below unity (0.72–0.64) (n = 6 for each category), demonstrating greater ribosome loading for the minor *10 allele, accounting for enhanced protein expression [24]. In contrast to OPRM1 (Fig 2A), the allelic *4/*10 was already strongly reduced in the monosome fraction, suggesting a main effect on first ribosomal loading, possibly *via* long-range interactions between the 3′ UTR *10 allele with the 5′ UTR.

#### C. Polysomal occupancy is not affected by the *ABCB1* (MDR1) rs1045642 (*3435T*) allele

We tested the effect of SNP *3435C>T* on mRNA expression and polysomal occupancy of ABCB1 mRNA in LCLs natively expressing ABCB1 and in HeLa cells transfected with an equal mixture of full-length ABCB1 expression plasmid with either *3435C* or *3435T*. Consistent with our earlier findings supporting enhanced mRNA turnover [24], the allelic mRNA ratio was 1.6 ± 0.1 (n = 6) in the cytosolic lysate, demonstrating a lower abundance of the minor allele *3435T*, presumably as a result of increased turnover. The allelic mRNA ratios did not significantly differ in the polysomal fractions (allelic ratio 1.64 ± 0.05 (n = 6) (Fig 2C). In *ABCB1* transfected HeLa cells, similar results were obtained, except that the allelic mRNA ratios were 1.3 ± 0.04 (n = 6) in total cytoplasm and remained the same throughout all gradient fractions (S2 Fig).

These results demonstrate the utility of detecting differences in allelic mRNA ratios between cytosol and polysomes.

### Polysome RNA loading in three LCLs measured with RNA-Seq

The purpose of this exploratory study was to determine the loading of all RNA classes onto polysomes, including mRNAs and noncoding RNAs, and their isoforms and alleles. Genetic effects can be indirect, for example by altering transcription start sites, splice sites, and polyadenylation sites leading to isoforms undergoing differential loading, or by altering sequestration into other cell structures such as P-bodies.

#### Polysomal loading of mRNAs and other RNA classes (>200 bp long)

RNA-Seq was performed on cytosolic extracts and polysomal fractions (3 ribosomes or more) from three LCLs. Previous studies have measured mRNAs in the heavy fractions of the sucrose gradient eluate; however, this fraction does not contain smaller particles that sequester RNA (e.g., RNPs), potentially further confounding isoform and allele distributions. For better comparison with widely available RNA-Seq profiles, we measured total RNA content in the cytosol, expecting to detect significant changes in RNAs sequestered on polysomes. As each RNA fraction was amplified (NuGen kit), and relative contributions from various RNA classes differed between cytosol and polysomes, the fraction of any given RNA species loaded onto polysomes was difficult to compare. Therefore, we focused on relative recovery of the various RNA classes and rank order of single RNAs compared to total RNA. The number of RNA-Seq reads per sample averaged ~40 million with a mean length of ~120 bases, of which ~70% aligned to annotated sequences representing mRNAs and various classes of noncoding RNAs including lncRNA and pseudogenes.

**Fig 3:**
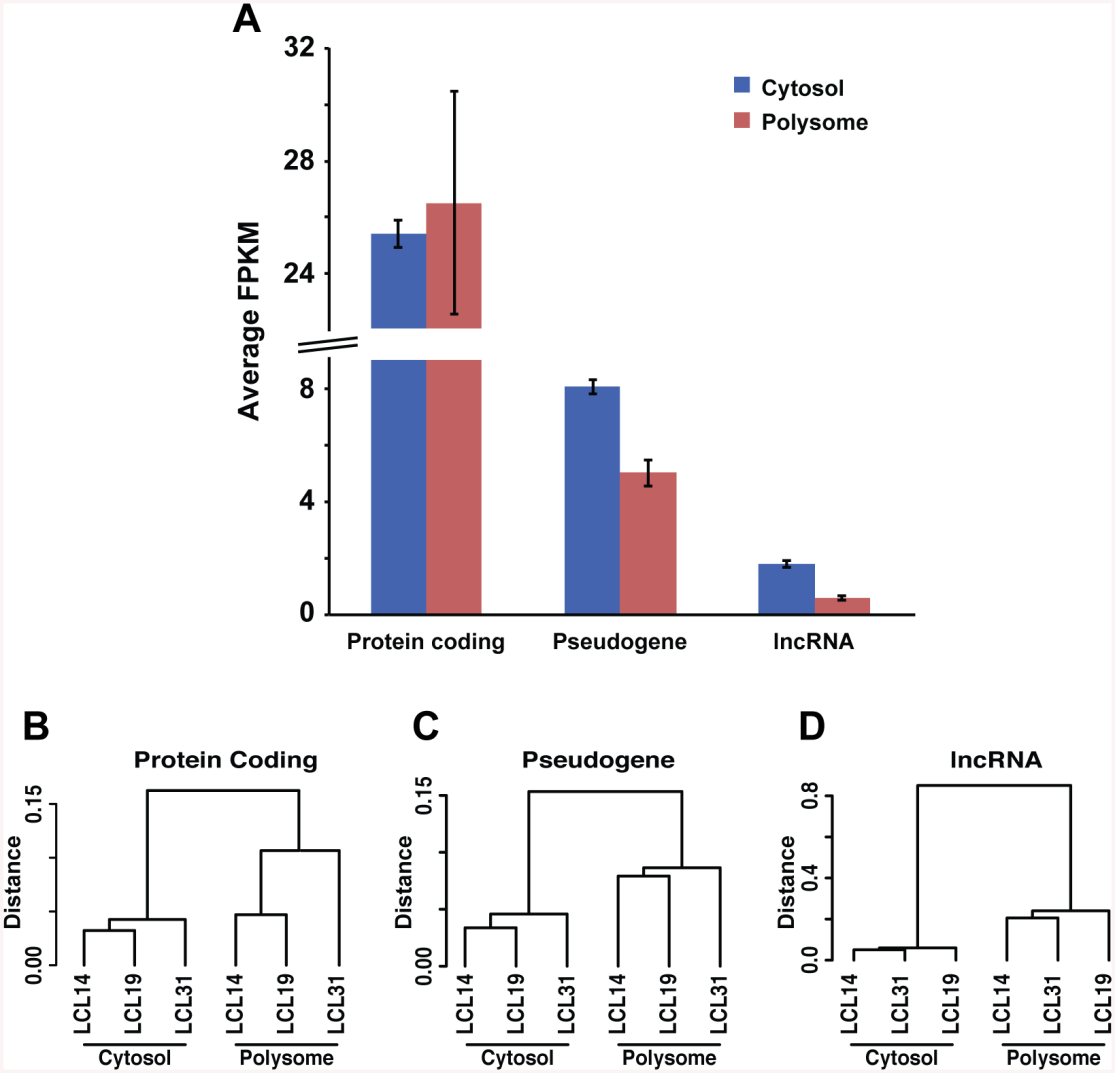
RNA classes in cytosol and polysomes from three LCLs, measured by RNA-Seq. After amplification of total cytosol RNA fraction and polysome fractions with NuGen, equal amounts of RNA were subjected to RNA-Seq, and sequence reads were aligned to annotated RNA to determine expression levels. **A.** Average expression level (normalized to FKPM) in cytoplasmic and polysome fractions from 3 different LCLs after exclusion of rRNA, tRNA, and mt-RNA. The pseudogenes and lncRNAs are reduced on polysome fractions. In cytosol lncRNA represent antisense-RNA (21.3%), lincRNA (4.7%) and lncRNA (74%). On polysomes the lncRNA distribution was drastically different: antisense-RNA (73%), lincRNA (14%) and lncRNA (13%). Error bars represent expression s.d. from 3 different LCL cells. **B-D**. Hierarchical clustering of the profiles of protein coding genes (B), pseudogenes (C) and lncRNA (D) performed with similarity indices (see Materials and Methods) for cytosol and polysome fractions. Observed difference of similarity indices between cytosol and polysome clusters is higher in lncRNA than in other RNA classes (note the difference on vertical scale).

The distributions of long RNA classes (>200 bases) within the cytosolic and polysomal fractions are shown in Fig 3A. While all RNA classes are represented on polysomes, individual RNAs among each class display large differences in their access to polysomes, consistent with polysomal loading as a critical step in regulation. Protein-coding mRNAs showing significant differences in relative abundance between polysomes and cytosol are listed in Table 1. Despite of low number of LCLs in this study relative to the large number of mRNAs tested, we were able to identify twenty-two mRNAs showing significantly different polysomal loading. Fourteen of these mRNAs, predominantly encoding histone proteins, showed robust enrichment on polysomes, reflecting the continuous demand for this type of protein. Among polysome-depleted genes, we observed mRNAs encoding MT-RNR2-like proteins, tumor protein translationally-controlled 1 (TPT1) and translation initiation regulators: polyadenylate-binding proteins 1 and 3 (PABPC1, 3). Possibly, these mRNAs reside mostly outside polysomes but can be rapidly recruited into polysomes when cellular conditions require rapid acceleration of protein synthesis.

**Table 1:** Protein coding mRNAs showing significant differences of polysome to cytosol ratios, adjusted p-value < 0.05.

Gene ID	Gene name	Polysome/Cytosol log2(fold change)
ENSG00000198327.3	HIST1H4F	2.9
ENSG00000188987.2	HIST1H4D	2.8
ENSG00000196226.2	HIST1H2BB	2.5
ENSG00000197409.6	HIST1H3D	2.3
ENSG00000124529.3	HIST1H4B	2.3
ENSG00000158406.2	HIST1H4H	2.1
ENSG00000196331.5	HIST1H2BO	2.1
ENSG00000197061.3	HIST1H4C	2.1
ENSG00000256018.1	HIST1H3G	2.0
ENSG00000187990.4	HIST1H2BG	2.0
ENSG00000160932.6	LY6E	2.0
ENSG00000198518.5	HIST1H4E	1.9
ENSG00000180573.8	HIST1H2AC	1.9
ENSG00000196532.4	HIST1H3C	1.8
ENSG00000070756.9	PABPC1	-1.9
ENSG00000151846.7	PABPC3	-2.0
ENSG00000133112.12	TPT1	-2.2
ENSG00000255823.1	MTRNR2L8	-2.9
ENSG00000255633.3	MTRNR2L9	-3.2
ENSG00000256045.1	MTRNR2L10	-3.3
ENSG00000256618.1	MTRNR2L1	-3.4
ENSG00000270672.1	MTRNR2L6	-3.4

While the polysome fractions contained high levels of protein-coding transcripts as expected, pseudogenes and long noncoding RNAs were also detected but at reduced relative levels compared to the cytosol (Fig 3A). Pseudogenes appeared to be more efficiently loaded onto polysomes compared to lncRNAs. Moreover, among the various RNA subclasses, large differences were observed. For example, lncRNA comprises antisense-RNA (21%), lincRNA (5%) and additional RNAs annotated in lncipedia as generic lncRNA (74%) in the cytosol, but on polysomes the distribution is drastically different: antisense-RNA (73%), lincRNA (14%) and lncRNA (13%). Pseudogenes and lncRNAs showing statistically significant polysome enrichment are listed in Table 2. For example, lncRNA lnc-EPHA6-1 was differentially enriched on polysomes and *U47924*.*28*, *CTD-2369P2*.*2*, and *RP11-16E12*.*2* showed similar trends. Their role in translation should be further studied. In contrast, RNA encoded by MT-RNR2-like 11, which is annotated as a pseudogene but nevertheless protein-coding, was significantly lower in the polysome fraction (log2 fold change -3.3, adjusted p-value 0.018), possibly accounting for the discrepant annotation. Polysome to cytosol ratio distributions of mRNA and ncRNAs for all tested genes are shown in S3A Fig mRNAs were significantly enriched on polysomes compared to cytosol.

**Table 2:** Noncoding RNAs showing significant differences of polysome to cytosol ratios, adjusted p-value < 0.05.

Gene ID	Gene name	Polysome/ Cytosol log2(fold change)	RNA type
lnc-EPHA6-1	lnc-EPHA6-1	-3.4	lncRNA
ENSG00000270188.1	MTRNR2L11	-3.3	Pseudogene
ENSG00000234782.2	RP11-442A13.1	-2.5	Pseudogene
ENSG00000214460.3	RP11-30P6.3	-2.5	Pseudogene
ENSG00000225471.2	RP11-262D11.2	-2.3	Pseudogene
ENSG00000217027.1	RP1-83M4.2	-2.1	Pseudogene
ENSG00000223361.5	FTH1P10	-2.0	Pseudogene
ENSG00000226221.1	AC022431.1	-1.9	Pseudogene
ENSG00000234009.1	RPL5P34	-1.7	Pseudogene
ENSG00000226948.1	RPS4XP2	-1.7	Pseudogene
ENSG00000220842.5	RP11-572P18.1	-1.7	Pseudogene
ENSG00000235552.4	RPL6P27	-1.7	Pseudogene
ENSG00000220793.4	RPL21P119	-1.7	Pseudogene
ENSG00000226608.2	FTLP3	-1.6	Pseudogene
ENSG00000223803.1	RPS20P14	-1.6	Pseudogene
ENSG00000243802.2	RP11-390K5.1	-1.6	Pseudogene
ENSG00000218175.2	AC016739.2	-1.6	Pseudogene
ENSG00000239559.2	RPL37P2	-1.6	Pseudogene
ENSG00000243199.1	RP11-408P14.1	-1.5	Pseudogene

To understand broadly the processes that govern the distribution of RNA classes, we determined similarity indices and constructed dendrograms of the three cytosolic and polysomal samples. Fig 3B–3D depicts the distance between branches of the dendrogram, calculated for expression profiles of various classes of RNA, revealing higher similarity of the profiles within cytosol and polysomes than between individual LCLs. lncRNAs displayed significantly greater diversity in profiles between cytosol and polysomes (Fig 3D, note different scales) compared to protein coding and pseudogene RNAs (Fig 3B and 3C), suggesting differences in the regulation of polysome loading between RNA classes, consistent with distinct overall functions.

#### Differential polysome loading of microRNAs

**Fig 4:**
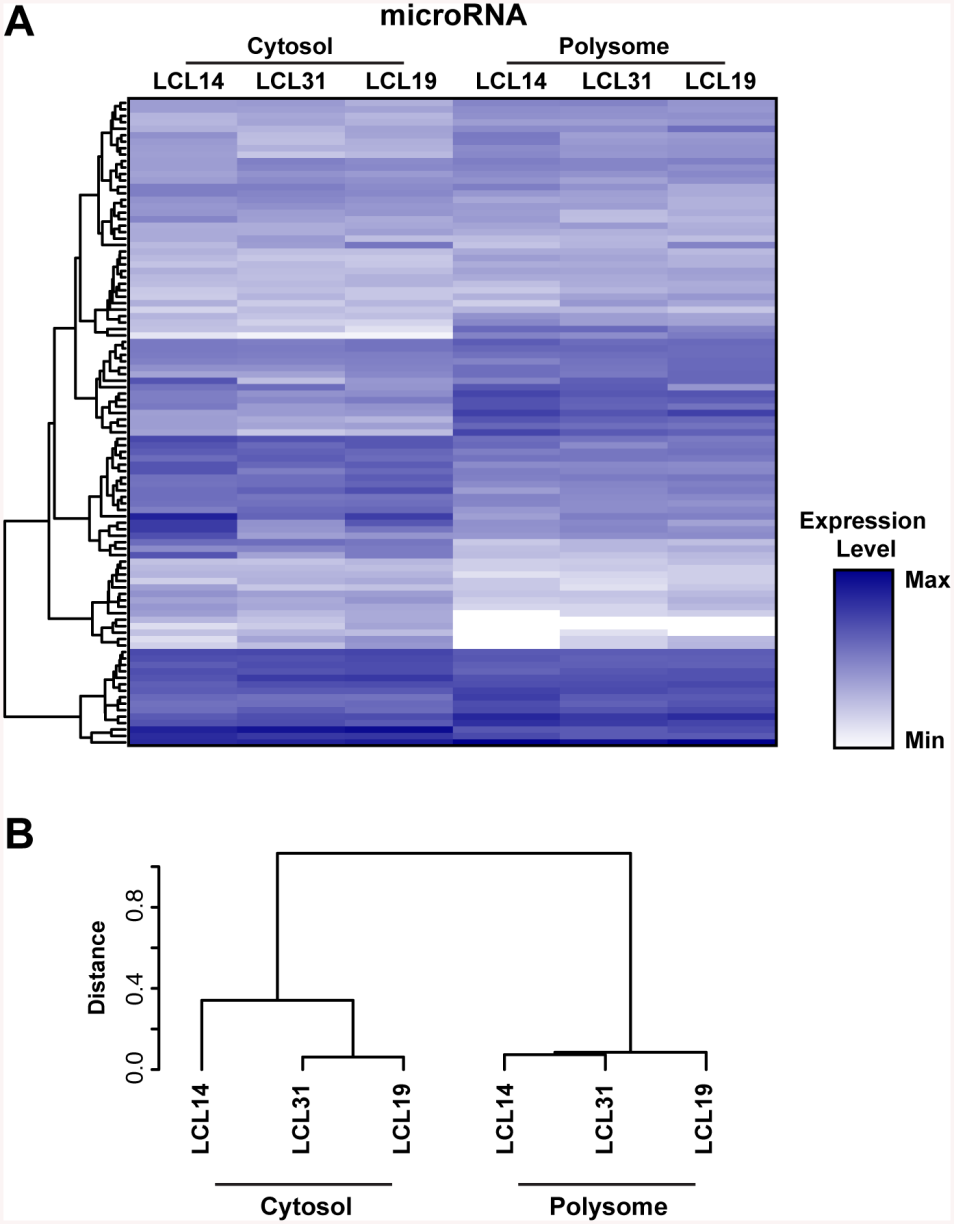
microRNA expression in cytosol and polysomal loading in three LCLs. **A.** Hierarchical clustering of microRNA expression profiles between cytosol and polysomes. microRNAs highly enriched on polysomes such as let-7a, mir-1275, and mir-145 had docking sites on mRNAs also enriched on polysomes. **B.** Hierarchical clustering of microRNA expression profiles measured with similarity indices (see Materials and Methods) reveal significant differences of microRNA profiles between cytosol and polysome fractions, showing reduced relative distances in comparison to cytosolic fractions.

We also sequenced short noncoding RNAs and focused our analysis on microRNAs. We observed a large number of microRNAs in the polysomal fraction, consistent with previous findings [27]. Table 3 lists the microRNAs with greatest differences between cytosol and polysomes, showing large enrichment or depletion of microRNAs on polysomes, suggesting specific functions, possibly in translation. Distribution of polysome to cytosol ratios for all detected microRNAs is shown in S3B Fig. Again, we performed clustering analysis, comparing microRNAs in both fractions (Fig 4A), highlighting the microRNAs consistently over- or underrepresented. The analysis of microRNA expression profiles, performed using Renyi’s divergence followed by hierarchical clustering, revealed substantial differences of microRNA profiles between polysomes and cytosol (Fig 4B). Specifically, the microRNA profiles of polysomes displayed higher similarity as compared to cytosol (Fig 4B), suggesting tight regulation of polysomal access as described previously [27].

**Table 3:** Top-scoring 20 microRNAs most strongly enriched on polysomes and most strongly polysome-depleted compared to cytosol, non-adjusted p-value < 0.05.

	Polysome/Cytosol ratio				Polysome/Cytosol ratio		
microRNA (enriched)	LCL14	LCL19	LCL31	microRNA (depleted)	LCL14	LCL19	LCL31
miR-340-3p	28	31	72	miR-21	0.12	0.10	0.08
let-7b	24	56	30	miR-1246	0.02	0.09	0.26
miR-181b	27	25	13	miR-155-3p	0.11	0.15	0.15
miR-423	13	18	15	miR-30b	0.15	0.20	0.24
miR-1275	11	12	4.1	miR-4301	0.12	0.18	0.32
miR-574	13	7.3	5.9	miR-181a-3p	0.09	0.32	0.23
miR-320a	6.8	8.3	9.2	miR-5100	0.06	0.45	0.12
miR-342	6.8	8.0	8.9	miR-1260b	0.12	0.30	0.24
miR-145	3.4	6.5	11	miR-551b-3p	0.14	0.32	0.28
miR-423-3p	6.7	5.1	8.3	miR-342-3p	0.21	0.27	0.30
miR-181c	3.5	6.9	4.8	miR-191	0.27	0.26	0.28
miR-181a	6.3	3.4	4.5	miR-106b	0.32	0.32	0.30
miR-193b-3p	3.4	3.1	7.0	miR-425	0.38	0.34	0.29
miR-877	1.9	4.2	6.9	miR-140-3p	0.40	0.24	0.37
let-7c	3.3	3.1	6.1	miR-625-3p	0.32	0.29	0.41
miR-1307-3p	4.6	4.1	3.3	miR-374b	0.39	0.29	0.41
miR-193b	3.5	2.9	5.4	miR-629-3p	0.27	0.55	0.31
miR-92a-3p	4.3	3.3	4.2	miR-27a-3p	0.39	0.34	0.42
let-7a	5.4	3.2	2.3	miR-4485	0.16	0.77	0.25
miR-7	3.5	4.4	2.3	miR-4448	0.19	0.29	0.76

Analysis of putative targets of the top 20 microRNAs with greatest preferential loading onto polysomes revealed 167 polysome-enriched mRNAs, with polysome/cytosol ratio ranging from 2 to 5.5 (non-adjusted p-value <0.01). Lymphocyte antigen 6 complex, locus E (LY6E), the putative target of mir-1275 (ratio >4), showed significantly higher polysomal occupancy (polysome/cytosol ratio 3.9, adjusted p-value 0.018). Polysome/cytosol mRNA ratio of *PIN1*, *RHOG*, *H2AFX*, and *NAT14* ranged from 2.8 to 3.8, (adjusted p-value 0.06 to 0.077), while corresponding microRNAs predicted to target these genes, mir-1275, let-7a (for *RHOG* and *NAT14*), and mir-145, had ratios from 2.3 to 12. These results support the model proposed by Molotski and Soen, in which microRNA occupancy on polysomes is determined by their interaction with target mRNA [27].

#### Differential loading of mRNA isoforms onto polysomes

**Fig 5:**
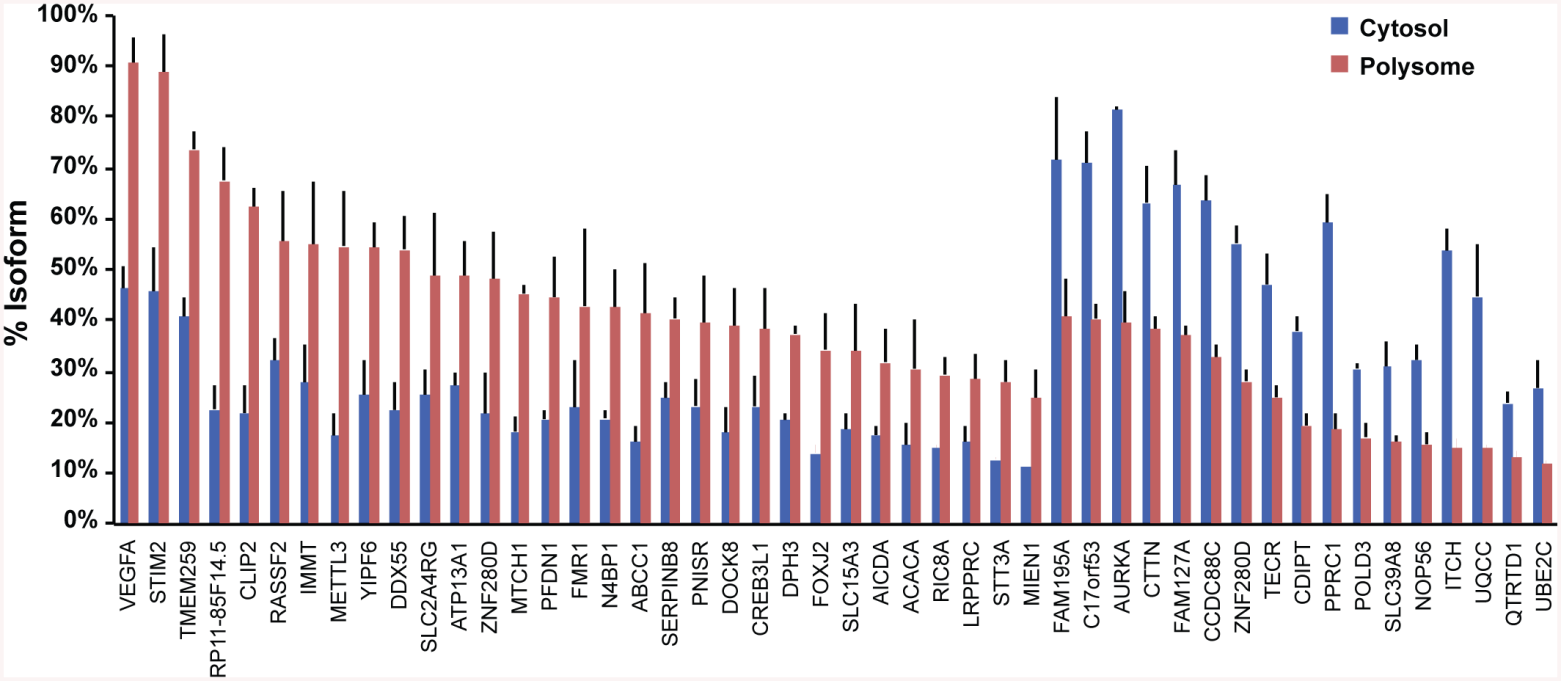
mRNA isoforms consistently different between cytoplasmic and polysome identified by RNA seq in three LCLs. Among all isoforms detected for a gene, some isoforms significantly differed in abundance between cytosol and polysomes. Isoforms showing similar pattern in all 3 LCLs are shown. Differences consistent among all cell lines suggest the possibility of isoform specific polysome recruitment. The results represent mean ± s.d. (n = 3). The isoforms are identified in S3 Table.

We then tested whether RNA isoforms, such as splice variants or RNAs with varying 3′ and 5′ UTRs, undergo differential loading. Determining mRNA isoforms from the RNA-Seq data yielded estimates of relative isoform distribution between cytosol and polysome fractions. RNA-Seq data on annotated isoforms were subjected to pairwise comparison between cytosol and polysomes, yielding 327 unique genes with major isoforms displaying different distributions (change in fraction of total mRNA >20% between cytosol and polysomes) (Fig 5). Select isoforms are listed in Table 4, separated into isoform RNAs enriched and depleted in polysomes. Genes generating polysome-enriched isoforms include *VEGFA*, *STIM2*, *CLIP2*, *IMMT*, *SLC24RG*, *FMR1*, *ABCC1*, and *SERPIND8*, whereas *FAM195A*, *AURKA*, *CTTN*, *ZNF280D*, *PPRC1*, and *SLC39A8* isoforms were depleted. Differences in isoform distribution are likely the result of distinct regulatory sequences. In addition, isoforms of annotated genes that do not contain open reading frames (ORFs), *e*.*g*., *ZNF280D* and *FAM127A*, were observed predominantly in cytosol, presumably lacking domains needed for polysomal loading.

**Table 4:** Genes expressing RNA isoforms with differential loading onto polysomes, and displaying different allelic RNA ratios in cytosolic and polysomal extracts. Targets with > 2 fold difference in allelic ratio between cytosol and polysome were analyzed for isoform differences. Results show both enrichment and depletion of isoforms on polysomes.

		% Isoform*		Allelic ratio			
Isoform ID	Gene	Cytosol	Polysome	Cytosol	Polysome	Transcript Type	Location
ENST00000226798.4	FRG1^a^	67	31	1	2.7	Protein coding	exonic
ENST00000253413.5	ATP6V1E1^a^	91	63	2.8	1.2	Protein coding	5' UTR/ 3' UTR
ENST00000254719.5	RPA1^a^	72	12	4.2	1	Protein coding	exonic/3' UTR
ENST00000285243.6	ANKRD40^b^	60	83	2.4	1	Protein coding	3' UTR
ENST00000292476.5	CPSF4^a^	99	73	2.6	1.1	Protein coding	intronic
ENST00000292538.4	CUX1^b^	38	61	1	2.2	Protein coding	exonic
ENST00000312419.3	POLD4^a^	90	63	2.9	1.3	Protein coding	3' UTR
ENST00000319004.5	GEMIN4^a^	91	75	1.2	3.8	Protein coding	exonic
ENST00000338754.4	TPST2^a^	76	12	2.8	1.2	Protein coding	exonic
ENST00000357984.3	TMUB2^b^	28	57	3.9	1.3	Protein coding	3' UTR
ENST00000361439.4	NSMCE1^b^	57	91	2.3	1	Protein coding	exonic/ 3' UTR
ENST00000361445.4	MTOR^a^	82	57	3	1.1	Protein coding	exonic
ENST00000368324.4	SYT11^a^	100	55	3.1	1.3	Protein coding	5' UTR
ENST00000372235.3	TMEM53^b^	27	59	2.8	1.1	Protein coding	3' UTR
ENST00000373616.5	MTCH1^a^	87	57	3	1.3	Protein coding	3' UTR
ENST00000398110.2	TPST2^b^	24	88	2.8	1.2	Protein coding	exonic
ENST00000440410.1	DLD^a^	55	30	2.5	1.1	Protein coding	exonic
ENST00000444411.1	MMS19^b^	39	62	2.2	1.7	Protein coding	exonic
ENST00000447062.2	WDTC1^a^	51	20	2.4	1	Nonsense mediated decay	3' UTR
ENST00000483469.1	GET4^a^	52	32	2.3	1.1	Retained intron	exonic
ENST00000483996.1	SUN1^b^	51	70	2.3	1.1	Retained intron	exonic
ENST00000491660.1	SERP1^b^	27	56	1.1	2.8	Protein coding	exonic
ENST00000492590.1	FHIT^a^	100	74	3.2	1.1	Protein coding	exonic
ENST00000492955.1	LY75^a^	55	30	4	1.8	Processed transcript	exonic/ intronic
ENST00000518016.1	FBXO16^b^	3	48	2.6	1.1	Retained intron	exonic
ENST00000529725.1	SIPA1^a^	55	33	2.8	1.3	Retained intron	exonic
ENST00000534474.2	MPV17L2^a^	65	39	3.4	1.1	Novel Protein coding	exonic
ENST00000540523.1	STAM^b^	52	71	1	2.6	Protein coding	exonic
ENST00000570487.1	RABEP1^b^	47	73	2.7	1.1	Protein coding	exonic
ENST00000587040.1	SYNRG^b^	38	58	2.6	1.5	Processed transcript	exonic

^a^RNA isoforms depleted on polysomes.

^b^RNA isoforms enriched on polysomes.

* Percentage of each isoform in the pool of all isoforms for given gene.

### Allelic RNA ratios in cytosol and polysomes of LCLs

Allelic differences in the cytosolic fraction result from regulatory variants altering gene expression, mRNA processing, and sequestration to cellular compartments [28], whereas differences in allelic ratios between cytosol and polysomes reflect differential allelic mRNA loading of the mRNAs or of their isoforms. We calculated allelic mRNA ratios for all RNAs with sufficient expression (>20 reads across a SNP) in the cytosol and polysome fractions of three LCLs as described [22]. Where available, we used several SNPs per transcript to determine mean allelic ratios and S.D. per transcript in one or more LCLs. This approach detected 630 genes in the cytosol and 559 genes in the polysomes with allelic RNA ratio ≥2-fold, with an approximate 25% overlap. RNAs with the highest allelic ratios are provided in S2 Table, including mRNAs and ncRNAs.

**Fig 6:**
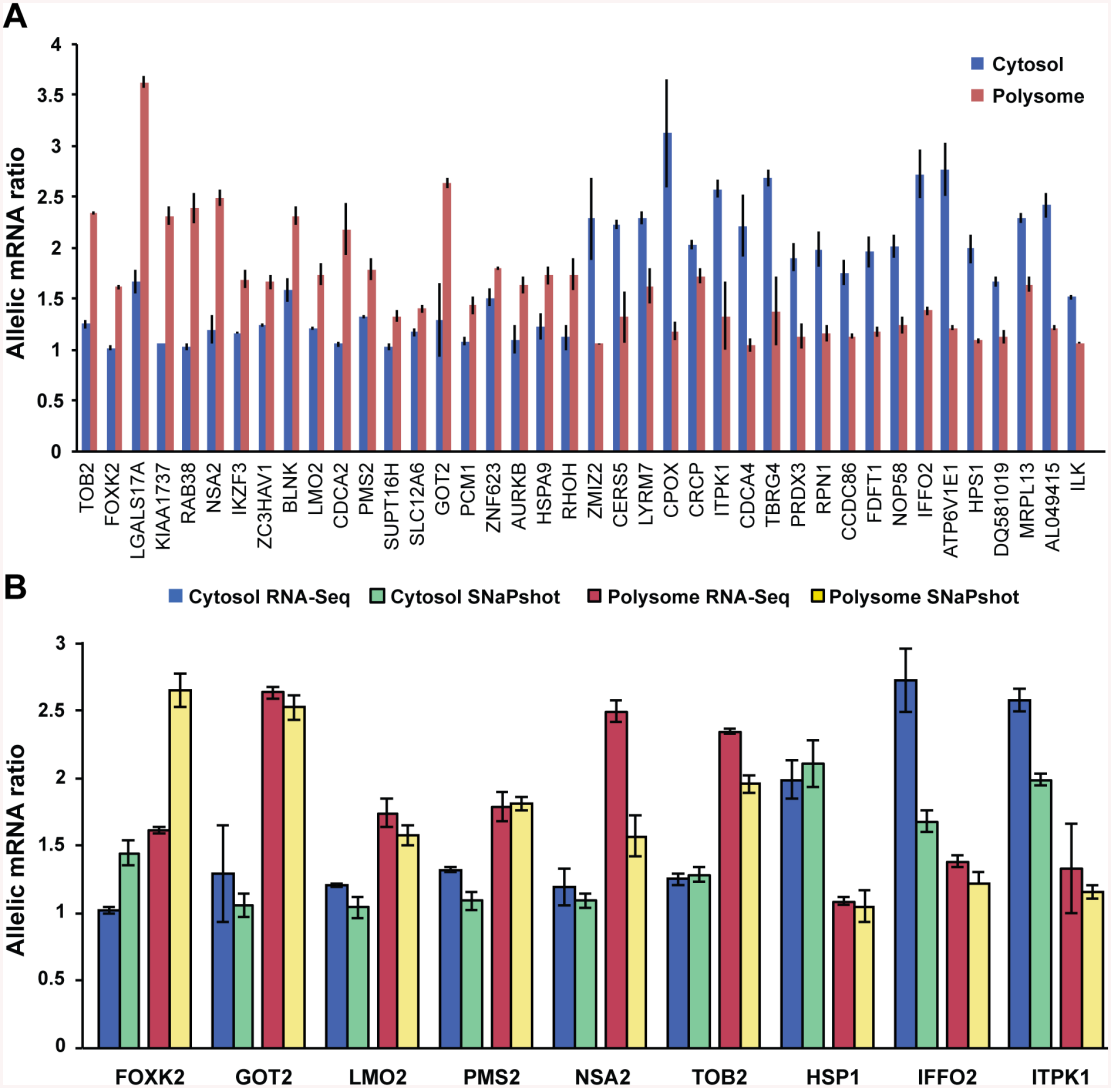
Allelic RNA ratios determined in whole transcriptome and translatome (polysomal RNAs) of three LCLs. **A.** Allelic RNA ratio differences measured in genes with two or more heterozygous SNPs are shown. To improve accuracy of allelic ratios measured using sequencing data, the ratios were measured at multiple SNPs of same transcript. **B.** Validation of mRNA allelic ratios obtained with RNA-Seq using SNaPshot assay. Both methods yielded similar allelic ratio differences between cytosol and polysomes.

To identify genetic factors altering polysomal loading, we searched for mRNAs showing ≥2-fold difference in the allelic RNA ratio (major/minor allele) between cytosolic and polysomal fractions of the same LCL sample (Fig 6). This approach yielded 60 mRNAs (11%) with the main allele enriched on polysomes compared to the cytosol, and 112 mRNAs (18%) with the main allele enriched in the cytosol (Fig 6A). To guard against false positive results, we selected mRNAs providing allelic ratio at more than one SNP (15%), shown in Fig 6A, some displaying large differences in allelic ratios between the cytosol and polysomes.

To validate allelic ratios calculated from RNA-Seq data, we employed a robust method (SNaPshot) [25] for accurate ratio analysis, selecting mRNAs with heterozygous states at two or more SNPs in the same LCL. Shown in Fig 6B, the results are consistent in detecting different allelic ratios between cytosol and polysomes for all test RNAs, even if the ratios in some samples were shifted to higher or lower values with SNaPshot (example *FOXK2*). *FOXK2*, *GOT2*, *LMO2*, *PMS2*, *NSA2* and *TOB2* mRNAs displayed a higher allelic ratio on polysomes, while *HSP1*, *IFFO2*, and *ITPK1* presented with a lower ratio on polysomes, as observed by RNA-Seq (Fig 6B).

Among the pseudogenes, *MGC70870*, and *GSTM2P1* had significant differences in allelic RNA ratios between cytosol and polysome fractions, suggesting similar processes regulating polysomal loading compared to mRNAs; possible translation into proteins should be evaluated. We also observed a few examples of robust allelic ratios deviating from unity in lncRNAs (*LINC00665*, *RP11-94L15*.*2*, *lnc-CTR9-3* (*ZBED5-AS1*)), but the ratios did not differ between cytosol and polysome, suggesting regulation of transcription or RNA processing. Overall, our results show that subcellular RNA samples have different allelic compositions, adding to the regulatory processes arising from genetic factors.

### Relationship between mRNA isoforms and measured allelic ratios in cytosol and polysomes

**Fig 7:**
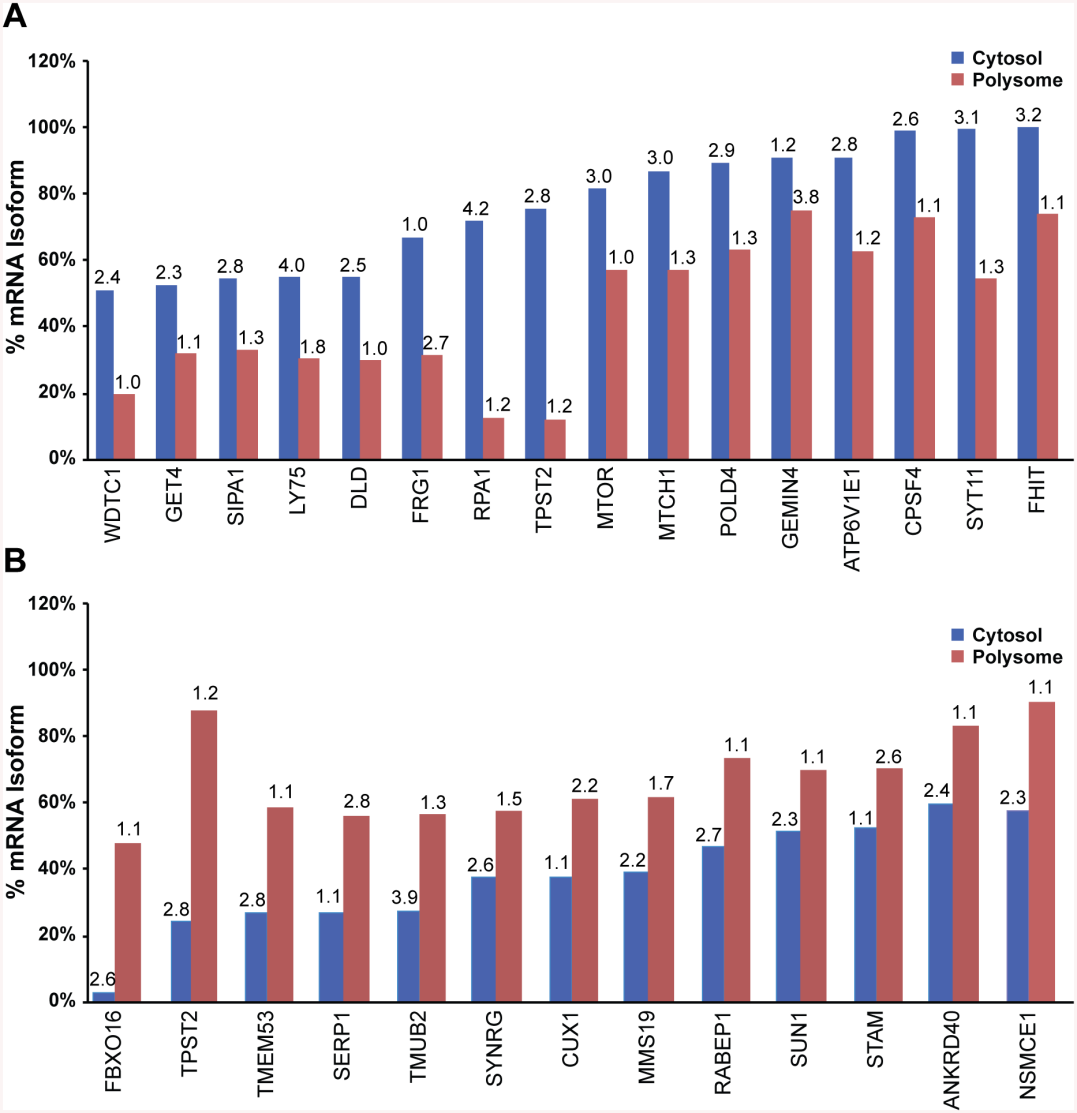
mRNA isoforms differentially loaded onto polysomes, and in addition displaying twofold different allelic RNA ratios (major/minor alleles) between cytosol and polysomes. **A.** Isoforms underrepresented on polysomes. **B.** Isoforms enriched on polysomes. Allelic mRNA ratios reflect the sum of all isoforms where the marker SNP is located. The allelic mRNA ratios are shown above the respective bar.

To distinguish effects of regulatory variants on isoform formation and on polysomal loading of isoforms, we surveyed all 327 genes with substantial differential polysomal loading of a major isoform (at least 20% difference between fractions relative to the total number of reads for transcripts at a gene locus) for allelic mRNA expression imbalance. Measured allelic RNA ratios can reflect contributions from different RNA isoforms if the marker SNP resides in an exon shared between them. For specific isoform analysis one needs to select SNPs in an exon not shared with other isoforms. Among genes with differential polysomal loading of isoforms, 82% displayed allelic ratios ≥2 fold in either cytosol or polysome in at least one LCL, but no significant difference between the two fractions, suggesting regulatory effects on transcription or RNA processing affecting all isoforms equally, for example equal changes in turnover rates. In contrast, 18% of mRNA with substantial isoforms detectable (>50% of total RNA expressed for each gene), for example *MTOR*, *POLD4*, and *FHIT*, displayed distinct allelic ratios between the cytosol and polysomes, suggesting a role in isoform formation or polysomal loading (Fig 7). The isoforms are identified in Table 4. Among isoforms depleted on polysomes, most have reduced allelic ratios of total RNA expressed a gene (major/minor allele) on polysomes (such as *WDTC1*, *SIPA1*, *LY75*, *DLD*, *RPA1*, *TPST2*, *MTOR*), except for *FRG1* and *GEMIN4* with higher polysomal allelic ratios (Fig 7A). A reduced ratio suggests that the minor allele isoform is enriched on polysomes. Similar results are observed with isoforms enriched on polysomes, again mostly showing reduced allelic mRNA ratios in polysomes (examples *TPST2*, *TMEM53*, *TMUB2*, *SYNRG*, *MMS19*), with only 3 isoforms having higher allelic ratios on polysomes (*SERP1*, *CUX1*, *STAM*) (Fig 7B). The interpretation of these results is hampered by allelic ratios at SNPs that could represent more than one mRNA isoform.

### Overlap between published eQTLs or GWAS associations and instances of differential allelic RNA ratios between polysomes and cytosol

A finding of allelic expression imbalance (AEI) signals the presence of regulatory variants. While distinct cytosol-polysome allelic RNA ratios likely arise from regulatory variants residing in the transcribed region of a gene locus, epigenetic effects and RNA editing are also possible [22]. As we have examined only three LCL, a single finding of AEI could implicate regulatory variants that could have a broad range of allele frequencies in the population, some showing high frequency. The instances of distinct AEI in polysomes reported here reveal a robust effect on RNA expression and presumably protein levels, with likely physiological consequences. Therefore, we searched for overlaps with GWAS associations (NHGRI GWAS Catalog) and published mRNA expression quantitative trait loci (eQTLs) from several databases, including Genotype-Tissue Expression project (GTEx), with expression data and eQTLs for multiple human tissues. In addition, Li et al. had identified polysomal eQTLs by measuring mRNA polysome recruitment with hybridization arrays in LCLs [7]. Table 5 summarizes overlaps with eQTLs for 37 genes identified here with strong allelic differences between polysomes and cytosol [29–57]. Strikingly, all but 4 of the 37 AEI genes also displayed eQTLs. Specifically, 11 genes had also been identified by Westra et al. [29]. Because such regulatory variants likely reside in the transcribed portion of the gene, we expected that in some cases the same SNP would be identified as an eQTLs as the one showing AEI, suggesting functional relevance. Indeed, in all 11 genes overlapping with the Westra study, all AEI SNPs were identical to eQTL SNPs (Table 5). Examples include *CDCA2* (rs6990278; minor allele frequency (MAF) = 0.19)–the regulator of chromosome structure during mitosis, mitochondrial ribosomal protein *MRPL13* (rs6650; MAF = 0.473), and RAS oncogene family member *RAB38* (rs1027027, MAF = 0.279). eQTLs in transcripts of genes such as nuclear proteins involved in regulation of proliferation and cell cycle, such as *NSA2* (rs6874609, MAF = 0.381) or ceramide synthase *CERS5* (rs7279, MAF = 0.201), were also identified by GTEx.

**Table 5:** Genes implicated as having genetic variants affecting polysomal loading (either directly or via RNA isoforms) were tested for associations with clinical phenotypes (all GWAS from dbGaP) and eQTLs.

		Allelic ratio				
Gene ID	Gene name	Cytosol	Polysomes	GWAS catalog	eQTL	References
ENSG00000154945.6	ANKRD40	2.4	1		**x**	[30]
ENSG00000131100.8	ATP6V1E1	2.8	1.2		**x**	[30]
ENSG00000122515.10	ZMIZ2	2.3	1.1		**x** *	[29]
ENSG00000184661.9	CDCA2	1.1	2.2		**x** *	[29]
ENSG00000170779.10	CDCA4	2.2	1		**x** *	[29]
ENSG00000139624.8	CERS5	2.2	1.3		**x** *	[29, 30]
ENSG00000080819.2	CPOX	3.1	1.2		**x** *	[29]
ENSG00000241258.2	CRCP	2	1.7	**x** *	**x** *	[29, 31]
ENSG00000079459.8	FDFT1	2	1.2	**x**	**x**	[30, 32, 33]
ENSG00000109536.7	FRG1	1	2.7	**x**	**x**	[30, 34]
ENSG00000125166.8	GOT2	1.3	2.6	**x**	**x**	[31, 35]
ENSG00000107521.14	HPS1	2	1.1		**x**	[30]
ENSG00000113013.8	HSPA9	1.2	1.7		**x** *	[29]
ENSG00000161405.12	IKZF3	1.2	1.7	**x** *	**x** *	[29, 36–43]
ENSG00000100605.12	ITPK1	2.6	1.3	**x**	**x**	[30, 44–46]
ENSG00000226025.5	LGALS17A	1.7	3.6	**x**	**x**	[47]
ENSG00000135363.7	LMO2	1.2	1.7	**x**		[48]
ENSG00000054219.9	LY75	4.0	1.8	**x**	**x**	[30, 49]
ENSG00000172172.3	MRPL13	2.3	1.6		**x** *	[29]
ENSG00000137409.14	MTCH1	3	1.3		**x**	[30]
ENSG00000198793.8	MTOR	3	1.1	**x**	**x**	[30, 50]
ENSG00000164346.5	NSA2	1.2	2.5		**x** *	[29, 30]
ENSG00000078674.13	PCM1	1.1	1.4	**x**	**x**	[30, 51]
ENSG00000122512.10	PMS2	1.3	1.8		**x** *	[29, 30]
ENSG00000123892.7	RAB38	1	2.4	**x**	**x** *	[29, 30, 52]
ENSG00000029725.12	RABEP	2.7	1.1		**x**	[30]
ENSG00000168421.7	RHOH	1.1	1.7	**x**		[53]
ENSG00000132383.7	RPA1	4.2	1		**x**	[30]
ENSG00000163902.7	RPN1	2	1.2	**x**	**x** *	[29, 54, 55]
ENSG00000213445.4	SIPA1	2.8	1.3		**x**	[30]
ENSG00000140199.7	SLC12A6	1.2	1.4		**x** *	[29, 30]
ENSG00000136738.10	STAM	1	2.6		**x**	[30]
ENSG00000006114.11	SYNRG	2.6	1.5	**x**	**x**	[30, 56]
ENSG00000136270.9	TBRG4	2.7	1.4		**x** *	[29]
ENSG00000183864.4	TOB2	1.3	2.3	**x**		[57]
ENSG00000105939.8	ZC3HAV1	1.2	1.7		**x** *	[29]
ENSG00000183309.7	ZNF623	1.5	1.8		**x** *	[29]

*The same SNP implicated in allelic RNA analysis in polysomes is also identified as a candidate variant in the databases.

Overlap between polysome AEI and clinical phenotypes in the database of Genotypes and Phenotypes (dbGaP) was observed in 16 of the 37 genes (Table 5). This finding suggests a link between regulatory variants and downstream phenotypes. In two genes, *CRCP* and *IKZF3*, the implicated dbGaP SNP was identical to the SNP identified by our AEI analysis, suggesting that it is the causative SNP or in high Linkage Disequilibrium (LD) with a functional variant.

We also searched for databases with protein eQTLs (pQTLs), but these are less well developed [58]. In one case, the level of GOT2, which showed polysome-specific AEI in this study, was associated with SNP rs7194417 [59]. Also showing allele selective polysome loading of one of its isoforms, IMMT had been identified in Battle’s study as having a pQTL [60].

## Discussion

Delineating the contributions of regulatory variants on protein translation is critical for understanding a substantial proportion of unresolved genetic regulation. The overall regulation of translation is considered a main factor determining protein abundance in cell lines [61], and improved correlations between polysomal mRNA and protein levels are better correlated to protein levels than total cellular mRNA levels [6]. Both findings together indicated that differential loading onto polysomes reflects a significant portion of translational regulation. By measuring allelic mRNA ratios with precision and accuracy, we demonstrate that one can detect differential loading of mRNA alleles onto monosomes and further progression into polysomes, as a means for uncovering *cis*-acting variants affecting translation. Here, we utilize two novel approaches for detecting the effect of genetic variants in mRNA on translational activity by measuring allelic mRNA ratios. The first approach to establish a proof of principle uses targeted expression of cloned constructs for *OPRM1*, *NAT1*, and *ABCB1* alleles [23, 24, 26], while the second utilizes endogenous expression in human cells to survey the entire polysome-bound transcriptome (*i*.*e*. the translatome) in LCLs and to assess the role of genetic variants in polysomal loading.

Our targeted gene results support the use of measuring allelic ratios during polysomal loading as a sensitive means for detecting genetic effects on mRNA translation, such as initial and subsequent ribosome loading, with *OPRM1* and *NAT1* and *ABCB1* as examples. Full sequencing of the transcriptome and translatome detects widespread differences in allelic ratios on translating mRNAs, providing evidence for *cis*-acting variants that influence translation. However, measuring allelic RNA ratios alone in short sequencing reads reduces the ability to attribute genetic effects to specific RNA isoforms, such as splicing or different transcription start site or poly-adenylation site usage, that occur before polysomal loading and yield RNA isoforms that then have different ability to load ribosomes. Importantly, determining allelic RNA ratios in polysomal fractions is less confounded by RNA isoforms present in other cellular fractions, while focusing on RNA alleles with likely functions in regulating translation. Therefore, differential allelic loading signals a *cis*-acting influence on translation, with likely biological consequences that remain obscured when measuring total cellular RNA content alone.

### Molecular genetics studies of target genes

The *OPRM1* non-synonymous polymorphism *118A>G* (N40D) had been shown to exert a dual effect on both overall mRNA expression levels and translational activity [23], while any effect on the protein’s function [62] remains uncertain. Upon co-transfection of full-length *OPRM1 118A>G* cDNA alleles, the main allele *A* is indeed more abundant in the cytosolic fraction, while it is further significantly enriched in the heavy polysome fractions, supporting previous findings of reduced expression and translation caused by *118G*. On the other hand only a small increase of the *A/G* ratio was observed in the monosome fractions, indicating that reduced loading of an initial ribosome onto the *G* allele mRNA was less robustly impaired compared to further ribosome loading. As the *118A>G* SNP resides towards the 5′ region of *OPRM1*, we speculate that the movement of the ribosome along the mRNA strand could be impaired, resulting in lower polysome loading.

The N-acetyltransferase allele NAT1*10 had been shown to increase expressed enzyme activity, compared to the wild-type (*4) which we had attributed to enhanced translational efficiency, presumably through the increased loading onto polysomes [24]. Here we have demonstrated in LCLs natively expressing *NAT1* that NAT1*10 does promote association of the mRNA allele with polysomes. Even though the *10 SNP is located in the 3′ UTR, loading of the first ribosome was already enhanced, in contrast to the observation with *OPRM1 118G*. 3′-untranslated regions (3′ UTR) appear to physically interact with the 5′ UTRs *via* protein complexes in regulating initiation of translation [63], with genetic variants modulating this process.

The synonymous SNP *3435C>T ABCB1* (rs1045642), (MDR1) has been widely shown to lower expression of the P-glycoprotein drug transporter. In LCL cells natively expressing MDR1, we confirm a lower level of expression of the *3435T* allele in the cytoplasmic lysate, consistent with enhanced mRNA turnover [25]. However, the allelic *C>T* ratios did not change in any of the polysomal fractions, which one would have expected if rare codon usage were to affect polysomal loading [26]. This result argues against ribosome stalling caused by *3435T* that leads to nonsense mediated mRNA decay or no-go-decay, and expected allelic ratio differences in polysome fractions. Further studies, such as ribosomal profiling [64, 65] will be needed to fully resolve the mechanism attributable to *3435C>T* underlying reduced MDR1 expression, a topic of considerable interest in predicting therapeutic drug response.

### Transcriptome analysis in polysomes

The RNA sequencing data provided here show relative distributions of different RNA categories and their occupancy on polysomes from three lymphoblast cell lines (Fig 3). Our approach provides a framework for functional studies of coding and non-coding RNAs and the impact of genetic factors on translatome dynamics. We find large differences between polysomal loading of various RNA classes and their isoforms (Table 1) as already reported. Overrepresentation of histone coding mRNA on polysomes might reflect high demand for DNA replication processes in cycling cells. In addition, our observation of pseudogenes and long noncoding RNA transcripts on polysomes is complimentary to a recent map of the human proteome [66], reporting peptides from many of these RNA categories are translated—blurring the boundaries between coding and non-coding genes. However relatively low representation of pseudogenes on polysomes suggests that members of this class predominantly function as noncoding RNA.

Differential loading of mRNA isoforms was observed for a number of genes (Fig 5, Table 4). In addition, non-coding RNAs generate isoforms that are differentially loaded on to polysomes (Table 2), suggesting different biological functions between their isoforms. Hierarchical clustering of RNA profiles in cytosol and on polysomes (Fig 3B–3D) illustrates systematic differences between cytosol and polysomes, and between individuals. The highest level of similarity was observed with lncRNA in cytosol extracts, suggesting a high level of regulation to maintain cellular equilibrium. In contrast, mRNA levels in cytosol varied most between LCLs.

Comparison of microRNAs in cytosol and polysomes showed differential association of microRNAs with polysomes suggesting distinct interaction with their target mRNAs (Fig 4A). microRNAs either accelerate degradation of mRNA or impede (or alter) translation. Several microRNAs were found to co-sediment with polysomes as seen in this study and others [27], suggesting an effect on translation. Hierarchical clustering of similarity indices suggests that microRNAs are tightly regulated in polysomes with little deviation between subjects (Fig 4B). The interactions between microRNAs and abundant mRNAs that encode microRNA docking sites has been proposed to determine polysomal loading of microRNAs [27], a finding reproduced here. Supported by the results in this study, the interplay between non-coding and protein-coding RNAs can be revealed by simultaneous RNA-Seq analysis of all RNA classes.

### Allelic RNA differences detected in transcriptomes on polysomes

Any deviation of allelic RNA ratios from unity (in autosomal genes devoid of copy number variants) reveals *cis*-acting regulatory factors altering polysomal loading or isoform expression, having downstream effects on isoform distribution onto polysomes. In addition, genetic variants can alter cellular trafficking by multiple mechanisms, such as microRNA binding. Any of these mechanisms can result in altered allelic ratios on polysomes, requiring further study to distinguish between them.

Transcriptome sequencing [22, 67] revealed variants covering protein-coding regions UTRs, ORFs, and noncoding RNAs, showing that allelic differences between cytosolic and polysomal mRNAs are more common than has been previously suggested by analysis of total RNA levels [7]. Therefore, regulatory variants affecting translation by differential polysome loading or isoform expression could be abundant [9].

Extensive localization of long noncoding RNAs to polysomes was reported earlier [68], but measuring the allelic ratio on polysomes to identify allele specific regulation had not been reported so far. Our findings provide a foundation for functional studies of the role of genetic variants and ncRNA in modulation of translation.

### Overlap with previous results and clinical relevance of genes showing differential allelic loading onto polysomes

We have surveyed GWAS databases (dbGaP) and RNA expression data (GTEx) to search for clinical associations and eQTLs in genes showing strong, differential allele-selective loading on polysomes (Table 5). This analysis revealed substantial overlap between the AEI SNPs and associated genes identified in this study, and both eQTLs and dbGaP hits (Table 5). It is remarkable that a finding of large AEI in a single sample can identify eQTLs, which require many more samples when measuring more variable mRNA levels [29]. In particular, the large Westra’s study was designed to detect trans-eQTLs specific to mRNA expression levels, but included numerous *cis-*eQTLs [29]. With use of only three LCLs, the majority of genes identified by our approach were also listed as polysomal eQTLs. This result strongly supports our allele-specific approach; however, in either study, an effect of variants on RNA isoform formation cannot be excluded. Application of the approach proposed in this study to a larger number of LCLs may lead to identification of novel regulatory variants of clinical relevance.

In a number of instances, the SNPs previously identified as eQTLs or GWAS hits were identical, supporting the notion that the SNP is causative or in high LD with a causative variant. Of particular interest are two overlapping AEI-GWAS SNPs. The *IKZF3* synonymous SNP rs907092 (MAF = 0.30) had been associated with primary biliary cirrhosis [36], and *CRCP* SNP rs875971 (MAF = 0.47, located in 3′ UTR) with aortic root diameter alternations [31]. In both cases the variant either is synonymous and does not change the amino acid sequence or is located in an untranslated region, yet both have robust effect on the extent of polysomal loading. Moreover, *IKZF3* is also associated with numerous other diseases including asthma and hay fever [69], rheumatoid arthritis [37], inflammatory bowel disease [39] and Crohn’s disease [42]. As an example of genes (rather than specific SNPs) implicated by allele-specific polysome enrichment, *LGALS17A* has been associated with obesity [47], and *RAB38* in sclerosis [52].

Upon completion of this study, a similar approach was recently published by Battle et al., employing RNA-Seq for mRNAs, ribosomal fraction of mRNA profiling and protein analysis, to identify eQTLs in mRNA associated with ribosomes, termed rQTLs, and pQTLs [60]. In this study, most eQTLs overlapped with rQTLs, while a number of unique pQTLs were found. The short sequence reads in ribosomal profiling in that study prevented analysis of RNA isoforms, shown here to play a critical role in polysomal loading. In addition, the study did not exploit allelic RNA ratios as a means to establish genetic effects on polysomal loading directly or on RNA isoform formation upstream. Ribosomal loading thus yields distinct results from polysomal RNA analysis.

In conclusion, the method developed in this study provides tools to explore functional genomics of translation and examine genetic loci linked to human disease. Using different cell or tissue types with this method is likely to reveal genetic effects vital for regulatory sequences. This approach allows us to prioritize genes showing strong *cis*-acting influence on protein translation for further functional studies. Our study provides a proof-of-principle that allelic effects on translation are pervasive and can be sensitively detected on polysomes, deployable on a transcriptome-wide scale using RNA-Seq to identify regulatory variants affecting translation.

## Materials and Methods

### Cell culture and transfection

Immortalized lymphoblastoid cell lines (LCLs; GM06994, GM06991, GM10852, GM07341, GM12250, GM13045, GM06991, GM12045, GM12043, GM10852) from the Utah Residents with European ancestry (CEPH) [70], were purchased from Coriell Institute for Medical Research. Human cervical cancer cells (HeLa), and Chinese Hamster Ovary cells (CHO) were obtained from American Type Culture Collection. Cells were cultured in DMEM (HeLa), DMEM-F12 (CHO), or RPMI1640 (LCLs), supplemented with 10% fetal bovine serum, 1% penicillin, and 1% streptomycin, in a humidified incubator at 37°C with 5% CO_2_. For RNA-Seq experiments, cells were grown to 5x10^5^ cells/mL density in T75 tissue culture flask and harvested. For transient transfections, 1.5×10^6^ cells were seeded into 10 cm^2^ culture plates and after 24 hours transfected with 1 μg plasmid solution containing equal concentration of two expression plasmids carrying either allele of a regulatory variants in a target gene, along with 75 ng emGFP as an internal control, using lipofectamine 2000 reagent (Life Technologies, Foster City, CA).

### Allelic mRNA ratio analysis of *OPRM1*, *ABCB1*, and *NAT1*

We used either transfection of expression plasmids (*OPRM1* (CHO cells), and *ABCB1* (HeLa cells) [67]), or natively LCL-expressed mRNA (*NAT1* and *ABCB1*). Cell lines were selected to match previous experiments with the same plasmids. *pcDNA3-OPRM1-118A* [23] was used to generate the *pcDNA3-OPRM1-118G* variant allele using Quick Change site-directed mutagenesis kit (Stratagene, La Jolla, CA) with primers *OPRM1-SDM-G118-F*, *OPRM1-SDM-G118-R* (S1 Table). The sequence was confirmed using *OPRM1-seq-primer-118-R*. Plasmid vectors expressing full length *ABCB1* (MDR1) *3435C* and *3435T* mRNA alleles were used as described [25]. *pcDNA6*.*2-emGFP* was used as a co-transfection control.

Cells were lysed and DNA was extracted from the nuclear pellet as described in [67]. RNA was isolated as described [67] from the total lysate solution (400 μL). An equal volume was layered onto a sucrose linear gradient (11 mL, 12 X 75 mm polystyrene, 10–50% w/v) and centrifuged at 228,000 x g in a Sorvall TH-641 rotor for 3.5 h at 4°C. Gradients were fractionated by continuous recording of UV absorbance at 254 nm into 24 fractions of approximately 0.5 mL, using a programmable density gradient fractionation system. Gradient fractions containing monosomes (80S ribosomes), light polysomes (gradient region containing 2–3 ribosomes per mRNA), intermediate (4–5 ribosomes per transcript) and heavy polysomes (6 and more ribosomes per transcript) were identified by corresponding UV peaks (Fig 1).

Eluant fractions corresponding to monosomes, light, intermediate, and heavy polysomes were collected. To each 200 μL sucrose gradient fraction, external control luciferase mRNA was added as an internal control, and RNA was isolated using trizol reagent (Life Technologies, Foster City, CA) and quantified using a Qubit 2 fluorometer (Life Technologies, Foster City, CA). RNA was isolated from every fraction followed by cDNA synthesis and qRT–PCR to study the sedimentation pattern of each target mRNA. For cDNA synthesis with oligodT priming (SSIII, Life Technologies, Foster City, CA), 0.5 μg RNA was subjected to *DpnI* (for plasmid transfections experiments only) and *DNaseI* treatment followed by real-time PCR quantification of ABCB1, OPRM1, NAT1, and luciferase mRNAs, using FAST-SYBR Green qRT-PCR supermix (Life Technologies, Foster City, CA), (for primers see S1 Table). The averaged cycle threshold (Ct) values were analyzed by a comparative Ct method [25] to obtain relative mRNA expression levels compared to luciferase control. For analysis of PCR amplicons by gel electrophoresis, extracts were amplified for 20–25 cycles to reflect the linear range during the SYBR green reaction in each experiment.

We tested allelic ratios at polymorphic sites using the primer extension method SNaPshot, (Applied Biosystems, Foster City, CA), as previously described [25]. qRT-PCR amplification of ABCB1, OPRM1, and NAT1 mRNA, and sequencing of the respective cDNA, was performed using PCR master mix (New England Biolabs, Ipswich, MA) as described [23–25, 67]. Samples were then subjected to electrophoresis on a 3730 Genetic Analyzer (Applied Biosystems, Foster City, CA) and evaluated with GeneMapper ID software V3.2 (Applied Biosystems, Foster City, CA) (Fig 1). In each sample, the allelic genomic DNA (gDNA) ratios were also measured, either of the cell’s genomic DNA isolated from the nuclear pellet, or of the transfected plasmid DNA isolated from cytosolic lysate. Allelic mRNA ratios between samples were then normalized to the DNA ratios set at 1.0. Results are from 3 independent experiments, each with 2 independent cDNA syntheses.

### RNA-Seq of LCL transcriptomes

#### Sucrose gradients for preparation of polysomes

Three LCL lines were obtained from the Coriell Institute: LCL14, LCL19, and LCL31. Cells were treated with 0.1 mM cycloheximide for 3 min, harvested, and washed with ice-cold PBS containing 0.1 mM cycloheximide, and harvested by centrifugation at 1000 RPM. The cell pellet was resuspended in 500 μL lysis buffer containing 150 mM NaCl, 50 mM Tris-HCl pH 7.5, 10 mM KCl, 10 mM MgCl_2_, 0.2% NP-40, 2 mM dithiothreitol, 2 mM sodium orthovanadate, 1 mM phenylmethylsulfonyl fluoride, and 80 units/mL RNaseOUT. After 10 min incubation on ice, samples were centrifuged at 7500 x *g* at 4°C for 10 min to pellet the nuclei (for gDNA analysis). 100 μL lysate was stored for the isolation of total cytoplasmic RNA and plasmid DNA. Genomic DNA was extracted by lysing the nuclear pellet. Equal volumes of total cytosol lysates (400 μL) were extracted for RNA analysis or subjected to sucrose gradient centrifugation as above.

RNA was isolated with Trizol reagent from the cytoplasm of the three LCLs (total cytosol content), and from pooled polysome sucrose fractions (>3 ribosomes; 250 μL). Two-step column purification and size separation were performed on each RNA sample. First, long RNA (>200 bases) was separated using SpinSmart RNA Purification column (Denville), and the flowthrough (<200 bases) was placed on a second column for small RNA (microRNA) separation with mirPremier microRNA isolation kit (Sigma-Aldrich, St. Louis, MO). RNA quality was evaluated on a Bioanalyzer.

Cytoplasmic (total) and polysome derived long RNA (25 ng each) was then converted to cDNA using the NuGen Ovation RNA-Seq System V2 (NuGen Technologies, San Carlos, CA), which uses both random hexamers and oligo-dT to amplify all RNA sequences, while suppressing cDNA formation from ribosomal RNA by >90%. ERCC RNA (External RNA Control Consortium, Ambion) controls were spiked into RNA prior to NuGen cDNA synthesis. The NuGen Ovation RNA-Seq kit produces non-stranded cDNA (3–5 microgram, measured with Qubit (Life Technologies, Foster City, CA)). The double-stranded cDNA derived from long RNAs was sheared to 150–200 bp fragments with a Covaris focused-ultrasonicator (Covaris, Inc. Woburn, MA) and recovered by centrifuging over an YM-30 spin filter (Amicon EMD Millipore Billerica, MA). Fragments longer than 100 bp were retained and eluted from the membrane.

#### Library preparation and cDNA sequencing using an Ion Torrent Proton instrument

We generated barcoded sequencing libraries from 100 ng of sheared cDNA using the NEBNext Fast DNA Library Prep Set for Ion Torrent sequencing (New England Biolabs, NEB, Ipswich, MA), as described [22, 71]. In a separate step, purified small RNA fractions (containing 100 ng RNA) were used for library construction using Ion Total RNA-Seq Kit v2 for small RNAs (Life Technologies, Foster City, CA). Pooled barcoded RNA libraries were sequenced on the Ion Torrent Proton platform (Life Technologies, Foster City, CA). In the small RNA fractions, only annotated microRNAs were studied further in this work.

#### Sequence alignment and mapping

RNA-Seq data were aligned using a two-step alignment approach (http://ioncommunity.lifetechnologies.com/docs/DOC-8434). Reads were initially mapped using tophat v2.11 [72]. Unmapped reads are extracted and realigned with bowtie v2 [73] using the 'local' and 'very sensitive local' options to allow clipping of the ends to improve the overall alignment score. Alignment files were sorted with samtools v1.19 [74] and merged with Picard (http://picard.sourceforge.net). Small RNA reads were aligned using miRanalyzer [75] with hg19 genome assembly-based bowtie index, and bowtie2 with index files of mature microRNA sequences. The effective library size was estimated using “DESeq” R package [76].

#### Estimation of RNA levels

Cufflinks v2.1.1 [77, 78] was implemented to estimate annotated RNA and isoform abundances, reported as Fragments Per Kilobase per Million reads (FPKM), normalizing the number of reads within a gene to the number of fragments per kilobase of exon and million mapped reads for a given sample. Expression measurements were quantified for transcripts annotated in GENCODE v18 [79] combined with additional transcripts present only in the lncipedia v2.0 non-coding RNA database [80]. These combined annotation sets capture a broad spectrum of protein coding and non-coding transcripts. Multi-read correction was applied to improve estimates when assigning multimapped reads to transcripts. In a first analysis, the sum of all RNA isoforms expressed from a given gene locus was compared between the cytoplasm (total) and polysome fraction and expressed as a ratio (fold-change) across the three LCL samples. Differential RNA levels between cytoplasmic total and polysomal fractions were determined with the *cuffdiff* application of Cufflinks [77], treating the three polysomal and cytosolic RNA-Seq samples as replicates.

#### SNP and allelic mRNA ratio detection

To make SNP calls, the mpileup pipeline by samtools v1.19 [74] was applied to each alignment file separately to maintain allele count estimates. Annotation was provided through ANNOVAR [81] detailing location within the gene based on UCSC gene annotations, implications of the SNP on protein-coding potential, and rs number based on dbSNP 135. For initial gene-wide allelic ratio estimates, SNPs were filtered based on SNP quality and an average mapping quality. Allelic mRNA ratios were estimated as previously described [22], applying a filter of 20 reads at any SNP as the minimum number for ratio analysis. A finding of allelic expression imbalance (AEI) was defined as a fold-change between the reference and variant alleles greater than 2X (in either direction). A difference in AEI between cytosol and polysomes was determined when the ratios differed twofold or more, accounting for cases where the minor allele is less abundant in the cytosol but more abundant in the polysomes (example major/minor allele in cytosol = 0.5, and in polysomes = 2; yielding a 4-fold difference), and *vice versa*.

### Data analysis

Statistical significance was expressed as p-values using the two-tailed student’s *t-*test for RNA-Seq and targeted experiments of ABCB1, OPRM1, and NAT1. The analysis was performed using Prism (GraphPad, San Diego, California, USA). Data are expressed as mean±SEM.

The non-parametric Mann-Whitney test (u-test) was used for statistical significance analysis of read counts of microRNA data. Pairwise analysis of microRNA sample similarity was performed using Renyi’s divergence calculated for each pair of samples. The pairwise similarity matrix was used for hierarchical clustering. Renyi’s divergence is a measure of similarity between two probability distributions. It is a generalization of the standard Kullblack-Leibler distance [82]. To obtain bounds on the sampling error, we applied computational methods based on the non-parametric (multinomial) bootstrap as described [82]. The analysis was performed using R package “divo”. The package is available for download in the CRAN repository (http://cran.r-project.org). Gene expression distributions of mRNA, pseudogenes, lncRNA, and microRNAs were analyzed using Chi-squared test. The analyses were performed using scripts written in R programming language [83].

## Supporting Information

S1 Fig mRNA distribution across polysome gradient and qRT-PCR detection of mRNA in pooled polysome fractions. **A-C.** RNA was isolated from sucrose gradient fractions (0.5 mL). Low cycle (20–25 cycles) RT-PCR was performed for each target RNA and the amplicons resolved on an agarose gel. **A.** OPRM1 mRNA transfected into CHO cells showed more mRNA in light and intermediate than heavy polysomes. **B-C.** NAT1 and ABCB1 mRNAs natively expressed in LCLs and HeLa cells, respectively, both showing increased levels in heavy polysome fractions. mRNP represent cytosolic mRNA either free or bound to ribonucleotide particles. **D-F.** Sucrose gradient fractions (0.5 mL) were pooled to reflect cytosol, monosomes, light, intermediate and heavy polysomes (Fig 1). qRT-PCR results were normalized to control mRNA added to samples prior to RNA isolation and fractionation. For details see Materials and Methods. **D.** *OPRM1* transfection into CHO cells. **E-F.** NAT1 and ABCB1 mRNA polysomes represent native expression in LCL cells, both showing increases towards heavy polysomes (lower Δ-Ct values).
(TIF)

S2 Fig ABCB1 allelic mRNA expression in cytosol and polysome fractions from transfected HeLa cells. ABCB1 *3435C>T* allelic mRNA ratios were measured in transfected HeLa cells expressing *ABCB1* 3435C and T alleles, demonstrating a significant reduction of the *3435T* allele to the same extent in all fractions. Data are representative of gradients done with extracts from 3 independent cultures (mean ± s.d., n = 6). **B.** qRT-PCR of pooled polysome ABCB1 mRNA shows increased occupancy on heavy polysomes (lower Δ-Ct values).
(TIF)

S3 Fig Distributions of polysome to cytosol ratios of RNA abundance. **A.** Comparison of distributions of polysome to cytosol ratios of RNA abundance between mRNA and noncoding genes revealed significant difference between means of the ratio frequency (p-value < 0.05). **B.** Distribution of polysome to cytosol ratios of microRNA abundance.
(TIF)

S1 Table List of primers used. (DOCX)

S2 Table RNAs with the highest allelic ratios. Allelic ratios were calculated with RNA-seq data of cytosol and polysome from LCLs, and 100 mRNAs and ncRNAs with highest allelic ratios in either cytosol or polysomes are listed. An allelic ratio 1.0 suggests equal expression of both alleles.
(DOCX)

S3 Table List of isoforms consistently different between all samples. For isoforms presented in Fig 5 (differences in isoforms based on 3 LCLs) and Fig 7 (based on different AEI ratios measurements in cytosol and polysomes of individual LCLs), this table provides isoform IDs.
(DOCX)
