# Supplementary figures and images for: Allele-Selective Transcriptome Recruitment to Polysomes Primed for Translation: Protein-Coding and Noncoding RNAs, and RNA Isoforms
Source: PLoS One. 2015 Sep 2;10(9):e0136798. doi: 10.1371/journal.pone.0136798 (PMC4558023; doi:10.1371/journal.pone.0136798)

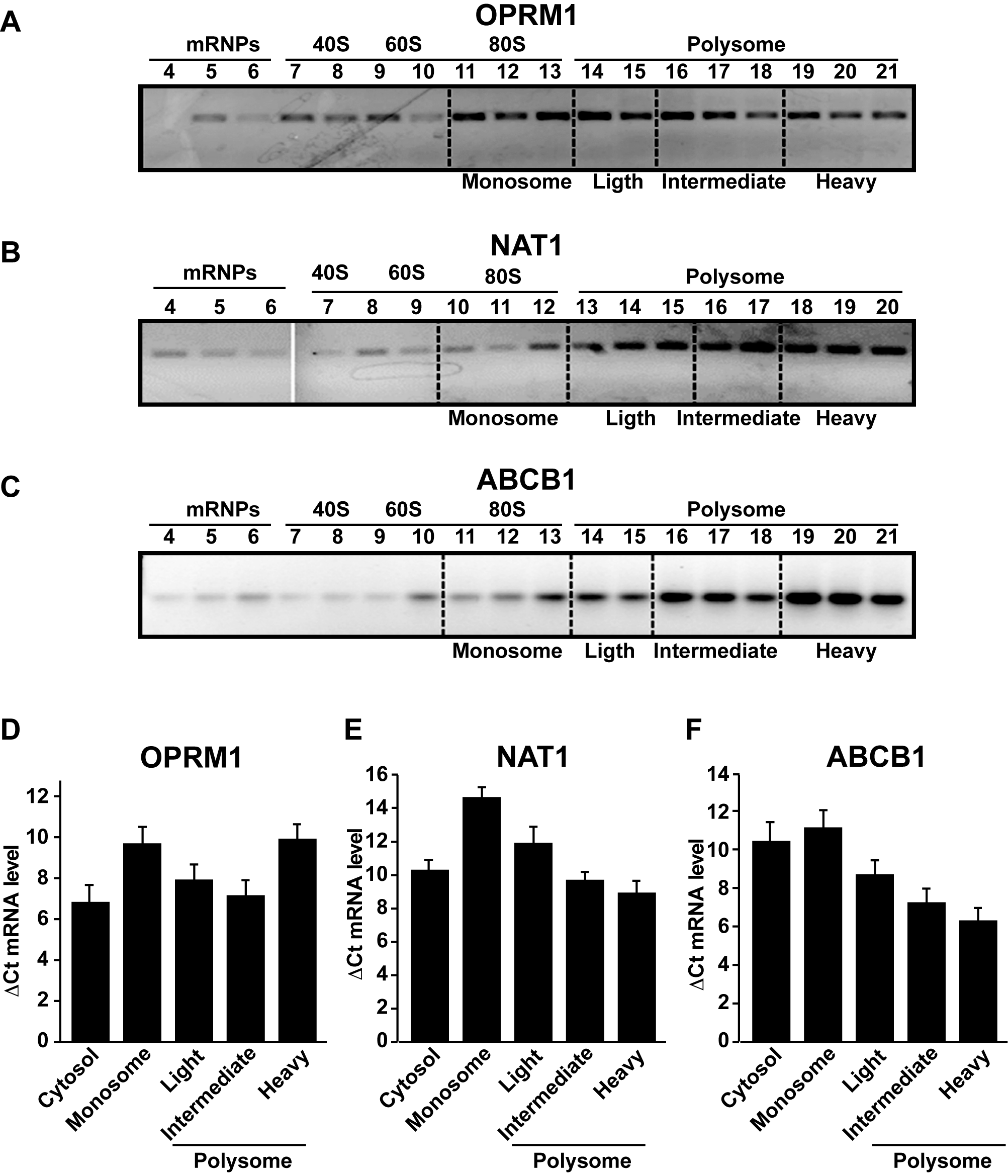

Supplement: S1 Fig — A-C. RNA was isolated from sucrose gradient fractions (0.5 mL). Low cycle (20–25 cycles) RT-PCR was performed for each target RNA and the amplicons resolved on an agarose gel. A. OPRM1 mRNA transfected into CHO cells showed more mRNA in light and intermediate than heavy polysomes. B-C. NAT1 and ABCB1 mRNAs natively expressed in LCLs and HeLa cells, respectively, both showing increased levels in heavy polysome fractions. mRNP represent cytosolic mRNA either free or bound to ribonucleotide particles. D-F. Sucrose gradient fractions (0.5 mL) were pooled to reflect cytosol, monosomes, light, intermediate and heavy polysomes (Fig 1). qRT-PCR results were normalized to control mRNA added to samples prior to RNA isolation and fractionation. For details see Materials and Methods. D. OPRM1 transfection into CHO cells. E-F. NAT1 and ABCB1 mRNA polysomes represent native expression in LCL cells, both showing increases towards heavy polysomes (lower Δ-Ct values). (TIF) [file pone.0136798.s001.tif]

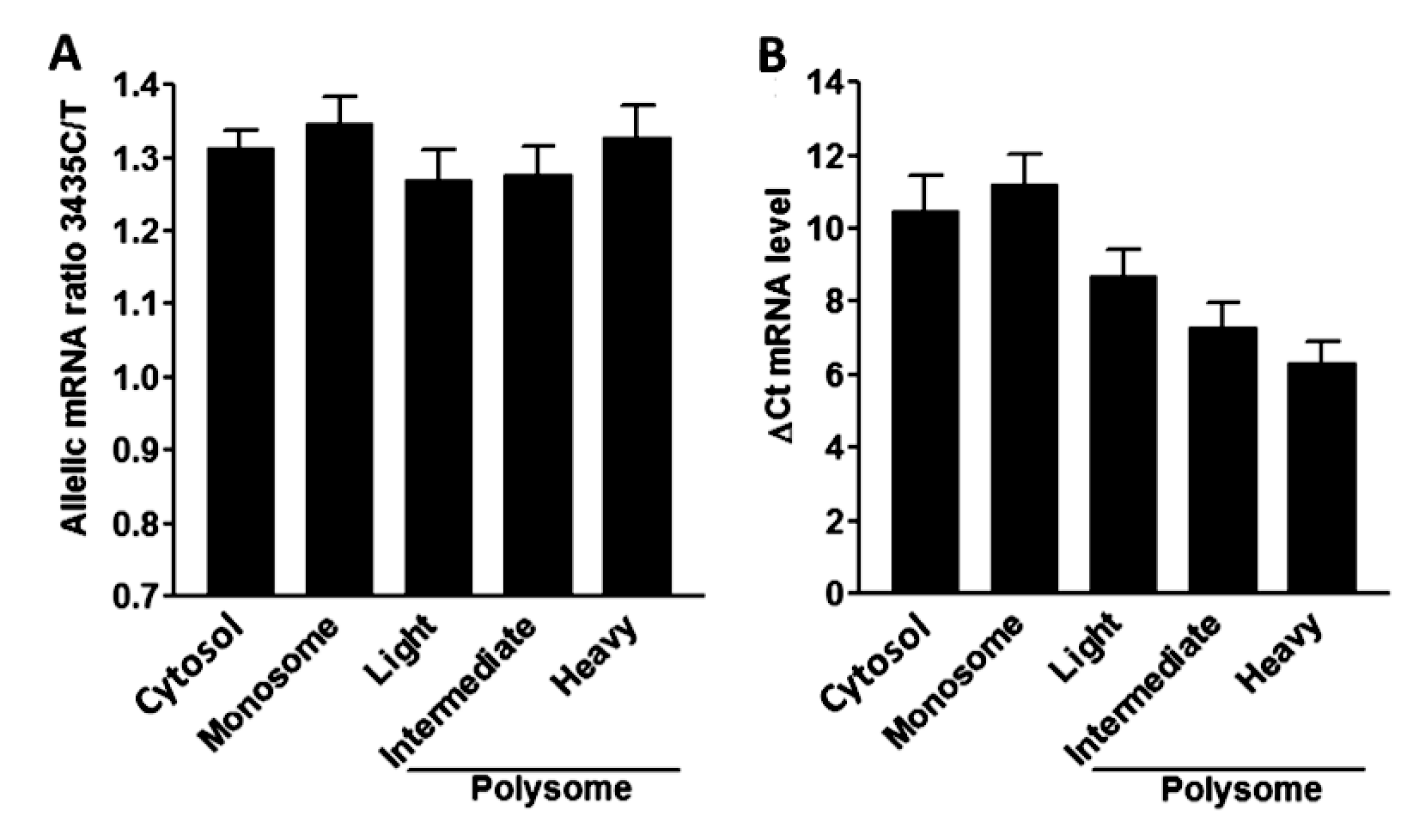

Supplement: S2 Fig — ABCB1 3435C>T allelic mRNA ratios were measured in transfected HeLa cells expressing ABCB1 3435C and T alleles, demonstrating a significant reduction of the 3435T allele to the same extent in all fractions. Data are representative of gradients done with extracts from 3 independent cultures (mean ± s.d., n = 6). B. qRT-PCR of pooled polysome ABCB1 mRNA shows increased occupancy on heavy polysomes (lower Δ-Ct values). (TIF) [file pone.0136798.s002.tif]

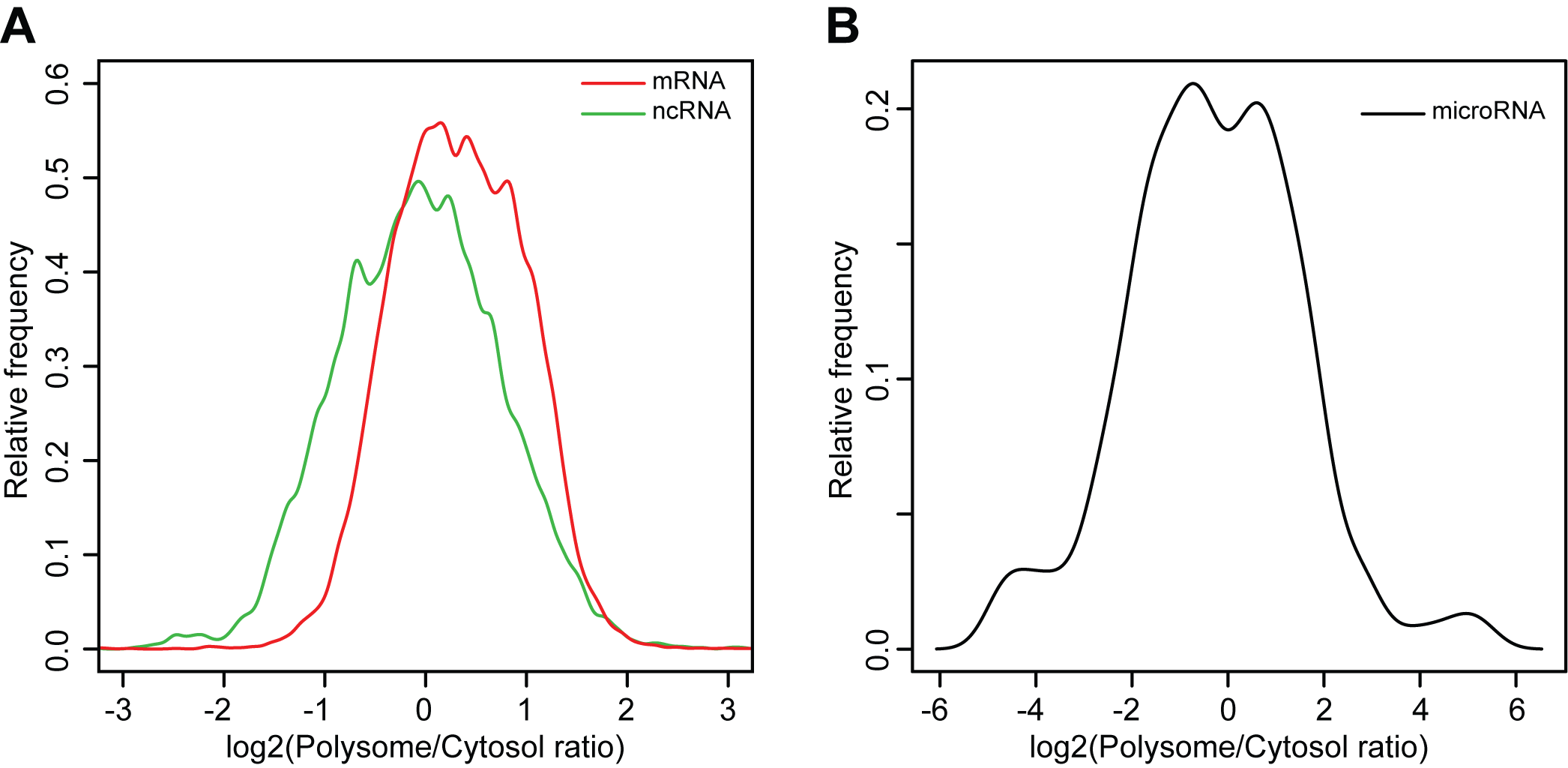

Supplement: S3 Fig — A. Comparison of distributions of polysome to cytosol ratios of RNA abundance between mRNA and noncoding genes revealed significant difference between means of the ratio frequency (p-value < 0.05). B. Distribution of polysome to cytosol ratios of microRNA abundance. (TIF) [file pone.0136798.s003.tif]
