# Supplementary material for: Allele-Selective Transcriptome Recruitment to Polysomes Primed for Translation: Protein-Coding and Noncoding RNAs, and RNA Isoforms
Source: PLoS One. 2015 Sep 2;10(9):e0136798. doi: 10.1371/journal.pone.0136798 (PMC4558023; doi:10.1371/journal.pone.0136798)
Supplement: S1 Table — (DOCX) [file pone.0136798.s004.docx]

**S1 Table. List of primers used.**

| **Name** | **Sequence 5′>3′** |
| --- | --- |
| ABCB1_3435_F | CCTATGGAGACAACAGCCGG |
| ABCB1_3435_R | GGCATGTATGTTGGCCTCCT |
| ABCB1_PE | CTCCTTTGCTGCCCTCAC |
| OPRM1_F | GGTTCCTGGGTCAACTTGTC |
| OPRM1_R | CAGGTCGGTGCGGTTC |
| OPRM1_PER | ACTGATCGACTTGTCCCACTTAGATGGC |
| OPRM1-SDM-G118-F | GTCAACTTGTCCCACTTAGATGGCGACCTGTCCGACCCATGCGG |
| OPRM1-SDM-G118-R | CCGCATGGGTCGGACAGGTCGCCATCTAAGTGGGACAAGTTGAC |
| OPRM1-seq-primer 118-R | GGATCCAGTTGCAGACATTGA |
| HTR2A_F | GTAATTCCACTCTGGACACAAACACT |
| HTR2A_R | AATTTTTTAGGCTGAAGGGTGAAG |
| HTR2A_PE | GCTTTGGATGGAAGTGCC |
| NAT1_*10_F | CTCACCAGTTATCAACTGACGAC |
| NAT1_*10_R | TTATTAGCCAACAATGTTTTAATATACTTT |
| NAT1_*10_PER | AACCACAGGCCATCTTTAAAA |
| emGFP_F | TCAAGGAGGACGGCAACATC |
| emGFP_R | TGTGGCGGGTCTTGAAGTTC |
| Luc_F | ATCGTGGATTACGTCGCCAGTCAA |
| Luc_R | TCTTTCCGCCCTTCTTGGCCTTTA |
| β-globin_F | ACATTTGCTTCTGACACAACT |
| β-globin_R | CTTGCCCCACAGGGCAGTAACG |
| Actin_F | GCTCACCATGGATGATGATATCGC |
| Actin_R | ATAGGAATCCTTCTGACCCATGCC |
