# Supplementary material for: Allele-Selective Transcriptome Recruitment to Polysomes Primed for Translation: Protein-Coding and Noncoding RNAs, and RNA Isoforms
Source: PLoS One. 2015 Sep 2;10(9):e0136798. doi: 10.1371/journal.pone.0136798 (PMC4558023; doi:10.1371/journal.pone.0136798)
Supplement: S2 Table — Allelic ratios were calculated with RNA-seq data of cytosol and polysome from LCLs, and 100 mRNAs and ncRNAs with highest allelic ratios in either cytosol or polysomes are listed. An allelic ratio 1.0 suggests equal expression of both alleles. (DOCX) [file pone.0136798.s005.docx]

**S2 Table. RNAs with the highest allelic ratios.**

| **Gene** | **Chr** | **Pos** | **Location** | **Cytosol AEI** | **Polysome AEI** |
| --- | --- | --- | --- | --- | --- |
| ABL1 | chr9 | 133761883 | 3′ UTR | 3.0 | 1.4 |
| ACP1 | chr2 | 272051 | exonic | 1.8 | 2.9 |
| AKAP11 | chr13 | 42876290 | exonic | 3.4 | 1.1 |
| ALKBH1 | chr14 | 78139988 | 3′ UTR | 3.2 | 1.7 |
| ALKBH5 | chr17 | 18112675 | 3′ UTR | 3.2 | 1.1 |
| APOL3 | chr22 | 36537893 | exonic | 3.4 | 1.2 |
| ATP5C1 | chr10 | 7830207 | exonic | 3.3 | 2.5 |
| BTBD2 | chr19 | 1997363 | exonic | 3.0 | 1.6 |
| C11orf24 | chr11 | 68030015 | exonic | 3.3 | 1.3 |
| C17orf81 | chr17 | 7163200 | 3′ UTR | 2.9 | 1.1 |
| CASP10 | chr2 | 202050677 | exonic | 2.9 | 1.9 |
| CCDC75(dist=5545), EIF2AK2(dist=3001) | chr2 | 37329283 | intergenic | 3.0 | 1.6 |
| CCDC91 | chr12 | 28412372 | exonic | 3.0 | 1.4 |
| CD226 | chr18 | 67531026 | 3′ UTR | 3.3 | 2.6 |
| CKS2 | chr9 | 91926124 | 5′ UTR | 3.8 | 1.2 |
| CLIP2 | chr7 | 73819191 | 3′ UTR | 2.9 | 2.0 |
| CNDP2 | chr18 | 72178161 | exonic | 3.1 | 1.6 |
| CPOX | chr3 | 98299365 | 3′ UTR | 3.1 | 1.2 |
| CR936688 | chr4 | 17629434 | upstream | 3.0 | 2.0 |
| CWC27 | chr5 | 64273018 | exonic | 1.1 | 2.9 |
| CYBRD1 | chr2 | 172412008 | 3′ UTR | 3.9 | 1.1 |
| DAP | chr5 | 10680487 | 3′ UTR | 3.5 | 1.9 |
| DDX6 | chr11 | 118621495 | 3′ UTR | 3.1 | 1.3 |
| DNMBP | chr10 | 101657880 | exonic | 3.0 | 2.8 |
| EI24 | chr11 | 125452313 | exonic | 3.0 | 1.5 |
| EPDR1 | chr7 | 37990955 | 3′ UTR | 3.6 | 2.3 |
| FHIT | chr3 | 59908126 | exonic | 3.2 | 1.1 |
| GFER | chr16 | 2035907 | exonic | 3.0 | 1.5 |
| GMNN | chr6 | 24780892 | exonic | 1.7 | 2.9 |
| IKZF3 | chr17 | 37917727 | 5′ UTR | 3.0 | 3.0 |
| GYG1 | chr3 | 148745236 | 3′ UTR | 3.7 | 1.5 |
| HELLS(dist=5887), CYP2C18(dist=75508) | chr10 | 96367743 | intergenic | 3.7 | 2.9 |
| HIST1H2AC | chr6 | 26124430 | 5′ UTR | 3.5 | 1.7 |
| HLA-B,HLA-C | chr6 | 31239108 | exonic | 3.2 | 1.9 |
| HSP90B1 | chr12 | 104341103 | exonic | 3.4 | 1.5 |
| IDI1 | chr10 | 1086116 | 3′ UTR | 3.3 | 2.1 |
| INPP5D | chr2 | 234054873 | exonic | 3.7 | 1.4 |
| INSR | chr19 | 7112645 | 3′ UTR | 3.3 | 2.7 |
| KCTD7 | chr7 | 66107971 | 3′ UTR | 3.1 | 2.8 |
| KDM3B | chr5 | 137754695 | exonic | 3.1 | 1.3 |
| KIF26B | chr1 | 245866020 | 3′ UTR | 3.0 | 1.8 |
| KIF3B | chr20 | 30922398 | 3′ UTR | 3.0 | 2.2 |
| KRR1 | chr12 | 75900588 | exonic | 3.0 | 2.2 |
| LIMS1 | chr2 | 109302388 | 3′ UTR | 3.1 | 2.5 |
| LOC390940 | chr19 | 44085827 | exonic | 3.2 | 2.4 |
| LOC729013 | chr11 | 10900584 | ncRNA  exonic | 2.9 | 1.2 |
| LY75,LY75-CD302 | chr2 | 160759491 | intronic | 4.0 | 1.8 |
| LYRM7(dist=17844), CDC42SE2(dist=40739) | chr5 | 130558963 | intergenic | 3.4 | 1.4 |
| MAN1A1 | chr6 | 119499566 | 3′ UTR | 3.0 | 2.1 |
| MAPK14 | chr6 | 36078180 | 3′ UTR | 3.8 | 1.1 |
| MED11 | chr17 | 4636565 | 3′ UTR | 3.8 | 1.1 |
| MPV17L2 | chr19 | 18307108 | exonic | 3.4 | 1.1 |
| MRPL52 | chr14 | 23299286 | exonic | 2.5 | 2.9 |
| MRPS17 | chr7 | 56023486 | downstream | 3.6 | 2.4 |
| MTCH1 | chr6 | 36936606 | 3′ UTR | 3.0 | 1.3 |
| MTOR | chr1 | 11181327 | exonic | 3.0 | 1.1 |
| MTPAP | chr10 | 30629226 | exonic | 3.4 | 1.4 |
| MVD | chr16 | 88718353 | 3′ UTR | 3.0 | 2.6 |
| NCR2 | chr6 | 41303592 | 5′ UTR | 3.6 | 1.3 |
| NLK | chr17 | 26518061 | exonic | 4.2 | 1.4 |
| NOTCH1 | chr9 | 139389184 | 3′ UTR | 3.3 | 1.3 |
| NUDT6 | chr4 | 123814308 | exonic | 3.0 | 2.7 |
| NUP214 | chr9 | 134020092 | exonic | 3.5 | 2.0 |
| PAFAH1B1 | chr17 | 2588717 | 3′ UTR | 1.2 | 2.9 |
| PDXP,SH3BP1 | chr22 | 38062589 | 3′ UTR | 3.6 | 1.1 |
| PGLS | chr19 | 17628587 | exonic | 2.9 | 1.7 |
| PHC3 | chr3 | 169806816 | 3′ UTR | 3.0 | 1.0 |
| PLBD2(dist=1493), SDS(dist=1300) | chr12 | 113828951 | intergenic | 4.0 | 1.5 |
| PNPO | chr17 | 46026156 | 3′ UTR | 3.3 | 1.7 |
| POLD4 | chr11 | 67119427 | 3′ UTR | 1.3 | 2.9 |
| POLE | chr12 | 133219831 | exonic | 3.5 | 2.3 |
| PRCP | chr11 | 82535963 | exonic | 3.2 | 1.5 |
| PRRC1 | chr5 | 126887706 | 3′ UTR | 2.9 | 1.2 |
| PRUNE | chr1 | 151006539 | exonic | 3.0 | 1.8 |
| PSMB6 | chr17 | 4699552 | exonic | 3.0 | 1.8 |
| PSME1(uc001wmg.3:  exon11:c.670-1G>T) | chr14 | 24607944 | splicing | 2.4 | 2.9 |
| RBM33 | chr7 | 155568550 | 3′ UTR | 3.3 | 1.1 |
| RHOH | chr4 | 40245990 | 3′ UTR | 3.3 | 2.2 |
| RNF111 | chr15 | 59368167 | exonic | 2.9 | 1.6 |
| RPA1 | chr17 | 1801189 | 3′ UTR | 4.2 | 1.0 |
| RPL13 | chr16 | 89630026 | 3′ UTR | 3.5 | 1.3 |
| RPL23AP82 | chr22 | 51223684 | ncRNA exonic | 3.6 | 3.1 |
| RPL36 | chr19 | 5690305 | 5′ UTR | 3.0 | 3.0 |
| RRAGD | chr6 | 90076685 | 3′ UTR | 3.8 | 2.7 |
| SAMD8 | chr10 | 76940216 | 3′ UTR | 3.6 | 1.1 |
| SELRC1 | chr1 | 53153432 | exonic | 3.3 | 1.1 |
| SERPINH1 | chr11 | 75283653 | 3′ UTR | 1.5 | 2.9 |
| SFT2D1 | chr6 | 166736362 | exonic | 3.1 | 1.6 |
| SLU7 | chr5 | 159835658 | exonic | 3.0 | 1.4 |
| SORL1 | chr11 | 121425955 | exonic | 1.2 | 2.9 |
| SRRD | chr22 | 26887829 | 3′ UTR | 3.6 | 2.0 |
| ST3GAL3 | chr1 | 44396646 | 3′ UTR | 3.7 | 1.5 |
| STARD3NL | chr7 | 38269668 | 3′ UTR | 3.8 | 1.4 |
| SYT11 | chr1 | 155829511 | 5′ UTR | 3.1 | 1.3 |
| TMPO | chr12 | 98943103 | 3′ UTR | 3.0 | 1.3 |
| UBC | chr12 | 125396680 | exonic | 3.6 | 3.2 |
| ZDHHC7 | chr16 | 85008366 | 3′ UTR | 3.1 | 1.1 |
| ZNF416 | chr19 | 58084930 | exonic | 3.0 | 3.2 |
| ZNF460(dist=10003), ZNF543(dist=16426) | chr19 | 57815439 | intergenic | 3.2 | 2.3 |

Allelic ratios were calculated with RNA-seq data of cytosol and polysome from LCLs, and 100 mRNAs and ncRNAs with highest allelic ratios in either cytosol or polysomes are listed. An allelic ratio 1.0 suggests equal expression of both alleles.
