# Supplementary material for: Allele-Selective Transcriptome Recruitment to Polysomes Primed for Translation: Protein-Coding and Noncoding RNAs, and RNA Isoforms
Source: PLoS One. 2015 Sep 2;10(9):e0136798. doi: 10.1371/journal.pone.0136798 (PMC4558023; doi:10.1371/journal.pone.0136798)
Supplement: S3 Table — For isoforms presented in Fig 5 (differences in isoforms based on 3 LCLs) and Fig 7 (based on different AEI ratios measurements in cytosol and polysomes of individual LCLs), this table provides isoform IDs. (DOCX) [file pone.0136798.s006.docx]

**S3 Table. List of isoforms consistently different between all samples.**

| **Gene** | **Isoform ID** |
| --- | --- |
| VEGFA | ENST00000520265.1 |
| STIM2 | ENST00000473519.1 |
| TMEM259 | ENST00000607316.1 |
| RP11-85F14.5 | ENST00000474250.1 |
| CLIP2 | ENST00000361545.5 |
| RASSF2 | ENST00000379400.3 |
| IMMT | ENST00000449247.2 |
| METTL3 | ENST00000298717.4 |
| YIPF6 | ENST00000374643.3 |
| DDX55 | ENST00000238146.4 |
| SLC2A4RG | ENST00000476221.1 |
| ATP13A1 | ENST00000291503.5 |
| ZNF280D | ENST00000560587.1 |
| MTCH1 | ENST00000373627.5 |
| PFDN1 | ENST00000514611.1 |
| FMR1 | ENST00000440235.2 |
| N4BP1 | ENST00000262384.3 |
| ABCC1 | ENST00000399410.3 |
| SERPINB8 | ENST00000542677.1 |
| PNISR | ENST00000498075.1 |
| DOCK8 | ENST00000474772.1 |
| CREB3L1 | ENST00000529193.1 |
| DPH3 | ENST00000488423.1 |
| FOXJ2 | ENST00000428177.2 |
| SLC15A3 | ENST00000544101.1 |
| AICDA | ENST00000537228.1 |
| ACACA | ENST00000588142.1 |
| RIC8A | ENST00000526104.1 |
| LRPPRC | ENST00000467058.1 |
| STT3A | ENST00000529886.1 |
| MIEN1 | ENST00000577810.1 |
| FAM195A | ENST00000307650.4 |
| C17orf53 | ENST00000245382.6 |
| AURKA | ENST00000371356.2 |
| CTTN | ENST00000415461.1 |
| FAM127A | ENST00000464369.1 |
| CCDC88C | ENST00000554165.1 |
| ZNF280D | ENST00000559352.1 |
| TECR | ENST00000215567.5 |
| CDIPT | ENST00000567459.1 |
| PPRC1 | ENST00000413464.2 |
| POLD3 | ENST00000530511.1 |
| SLC39A8 | ENST00000356736.4 |
| NOP56 | ENST00000492135.1 |
| ITCH | ENST00000535650.1 |
| UQCC | ENST00000374377.5 |
| QTRTD1 | ENST00000493014.1 |
| UBE2C | ENST00000372568.4 |
| WDTC1 | ENST00000447062.2 |
| GET4 | ENST00000483469.1 |
| SIPA1 | ENST00000529725.1 |
| LY75 | ENST00000492955.1 |
| DLD | ENST00000440410.1 |
| FRG1 | ENST00000226798.4 |
| RPA1 | ENST00000254719.5 |
| TPST2 | ENST00000338754.4 |
| MTOR | ENST00000361445.4 |
| MTCH1 | ENST00000373616.5 |
| POLD4 | ENST00000312419.3 |
| GEMIN4 | ENST00000319004.5 |
| ATP6V1E1 | ENST00000253413.5 |
| CPSF4 | ENST00000292476.5 |
| SYT11 | ENST00000368324.4 |
| FHIT | ENST00000492590.1 |
| MPV17L2 | ENST00000534474.2 |
| FBXO16 | ENST00000518016.1 |
| TPST2 | ENST00000398110.2 |
| TMEM53 | ENST00000372235.3 |
| SERP1 | ENST00000491660.1 |
| TMUB2 | ENST00000357984.3 |
| SYNRG | ENST00000587040.1 |
| CUX1 | ENST00000292538.4 |
| MMS19 | ENST00000444411.1 |
| RABEP1 | ENST00000570487.1 |
| SUN1 | ENST00000483996.1 |
| STAM | ENST00000540523.1 |
| ANKRD40 | ENST00000285243.6 |
| NSMCE1 | ENST00000361439.4 |

For isoforms presented in Fig. 5 (differences in isoforms based on 3 LCLs) and Fig. 7 (based on different AEI ratios measurements in cytosol and polysomes of individual LCLs), this table provides isoform IDs.
